# Intentional and actional components of engaged participation in public health research studies: qualitative synthesis of a recruitment and retention process into the theory-informed INTACT-RS framework

**DOI:** 10.1186/s12874-023-01838-3

**Published:** 2023-01-16

**Authors:** Jonas Lander, Andrea Heiberger, Julia Von Sommoggy, Anja Alexandra Schulz, Carolin Dresch, Hala Altawil, Gwendolyn Schmitt, Markus Antonius Wirtz

**Affiliations:** 1 Institute for Epidemiology, Social Medicine and Health Systems Research, Hannover Medical School (MHH), Carl-Neuberg-Str. 1, 30625 Hannover, Germany; 2 Research Methods in Health Sciences, Faculty for Mathematics, Natural Sciences and Technology, University of Education Freiburg, Freiburg, Germany; 3 Medical Sociology, Department of Epidemiology and Preventive Medicine, University of Regensburg, Regensburg, Germany; 4 Public Health & Health Education, Faculty for Mathematics, Natural Sciences and Technology, University of Education Freiburg, Freiburg, Germany

**Keywords:** Recruitment, Recruiting, Retention, Study participation, Public health, Framework

## Abstract

**Background:**

Ensuring motivated and successful study participation is a key challenge in the design and conduct of health research studies. Previously, recruitment barriers and facilitators have been identified mainly from experience, and rarely based on theoretical approaches. We developed a framework of intentional and actional components of engaged participation in public health research studies (INTACT-RS), informed by psychological behavioral models. We aimed a) to identify precise indicators for each framework component and b) to better understand which components and decision processes are essential for study participants.

**Methods:**

Within a multicenter research network, we applied various approaches to recruit parents of newborns, pediatricians, and midwives. All recruitment processes were documented from the perspective of both participants and researchers. We used different qualitative and quantitative data material, which we applied in a multistage process according to the basic principles of qualitative content analysis.

**Results:**

INTACT-RS encompasses pre-intentional, intentional and actional phases with a total of *n* = 15 components covering all aspects of an individual’s involvement with a research study. During intention formation, an understanding of efforts and benefits, why participation is valuable beyond contributing to research, and how others perceive the study, were particularly important to (potential) participants. Subsequently (intentional phase), participants consider how and when participation is compatible with their own resources, ability and availability, and hence seek for close communication with, and flexibility and support from the research team. During and after (initial) participation (actional phase), participants’ assessment of whether expectations and interests have been met impact crucial further steps, especially the willingness to continue and to recommend participation to others. A strong topic-wise and or supportive participation interest as well as active, continuous exchange with the researchers appeared to be central determinants of study completion and data validity.

**Conclusions:**

A theoretical framework is now available to plan and conduct recruitment of different target groups, which accounts for essential motivational and volitional decision-making processes. Based on empirically specified constructs, possible barriers can be addressed even before the initial recruitment process. Therefore, recommendations for scientific practice have been formulated.

**Supplementary Information:**

The online version contains supplementary material available at 10.1186/s12874-023-01838-3.

## Introduction

Successfully recruiting study participants and ensuring their motivated participation is a key challenge in planning and conducting health research studies [1–3]. In clinical trials, recruitment often occurs through direct patient contacts, calls for participation by clinicians, or pre-existing databases. However, previous findings indicate that although these measures can improve participation, reaching an adequate sample size and representativeness remains difficult [4, 5]. In public health research, a range of options for recruitment are available, too, but limited resources– particularly in smaller, qualitative research projects – or the lack of direct benefits for participants make this process challenging [6, 7].

Unsurprisingly given the significance of this topic, there is a considerable body of research about the design and organization of recruiting processes: Various systematic and scoping reviews have summarized barriers and facilitators based on individual descriptions of what works and what does not [5, 8–10]. Other studies have reviewed specific methods, e.g., the use of social media for recruitment [11–13], or the development and application of recruitment incentives [14]. Others again have directly assessed target groups’ perspectives on recruitment, mainly trialists and principal investigators [15], doctors and other healthcare professionals (HCPs) [16–19], and lay people [20–22]. Further studies have analysed recruitment strategies qualitatively and quantitatively, looking at recruitment data retrospectively [23] and statistically [24, 25], reflecting on self-experienced recruitment barriers [26], and describing the use of modified recruitment processes [27].

Lastly, some studies suggest how recruitment could be improved, by tabular summaries of identified recruitment facilitators [17, 24, 28], structured recommendations for the recruitment process [16, 29], and ‘principles’ and ‘frameworks’ for specific aspects, e.g. sampling framework [30] and recruitment maximization [23]. A Cochrane review provides a “Will I take part” conceptual model, which, based on qualitative evidence synthesis, describes five decisive determinants (effective trial communication, feeling nothing to lose, chance to help others, feeling something to gain, encouragement of other people). The authors also provide a comprehensive list of questions recruiters and trialists can consider when planning for recruitment, e.g., “will trialists aim to minimize time commitments?” and “will trial information be delivered verbally with face-to-face contact?” [5].

These and related contributions seem first and foremost to draw their conclusions from the perspective of researchers based on current recruitment practice. The aim of our study is to take the perspective of potential study participants who are considering taking part in the study. For them to make a decision, their attention must be aroused, and they must develop interest and motivation to participate, based on the subjectively expected effort and benefit. Therefore, explicit clarification of what is important and persuasive to people is needed to elicit engagement, and there needs to be a better understanding of psychological processes determining how individuals decide. Thus, a theoretical basis is missing to describe decision-making processes of potential participants and to explain their influence on engaged study participation. This clarification is essential to develop target group-specific recruitment strategies. Further, previous research has shown that those who are intrinsically motivated participate with greater concentration and invest more time in their participation [31]. Hence, interested and motivated participation may be essential to the validity of collected research data [32, 33].

### Theoretical framework

When the opportunity of participating in a study catches an individual’s attention, appraisal and consideration processes begin that determine whether the person decides to participate in the study. First, according to behavioural models, a phase of intention formation must be assumed (pre-intentional or motivational phase) [34, 35]. According to Bandura’s [36] social–cognitive theory, confidence in one’s ability to handle the demands of participation is essential. Furthermore, interested study participants pursue individual goals when considering participation, for example receiving a gratification, gaining knowledge, or helping to advance health care or health science. This results in a cost-benefit analysis in which positive consequences of participation should exceed anticipated efforts.

During intention formation, interest in study participation is crucial. Interest exists when one pays attention to an object to which one ascribes a subjective value and which is significant to personal needs. The more participation experience is considered significant and epistemic needs are expected to be fulfilled, the higher the interest in and commitment to a study. According to Holland [37], interest in study participation can be *intellectual*/*investigative* (curious, knowledge-oriented), *realistic/practical* (trying out spontaneously), *conventional* (confidence in the credibility of the study), *entrepreneurial* (related to social and financial gratification), *experiential* (contact/exchange with other people), or *social–helpful* (support of research/researchers). Note that the first three categories are broadly motivations intrinsic to the study, and the last three extrinsic – which means to consider study participation for reasons other than the subject matter, e.g. participating because of an incentive. Krapp [38] emphasizes that intrinsically-motivated interest seeks to establish and extend a relationship with or acquire and deepen knowledge about an object.

Based on Heckhausen (1989) and Schwarzer (1992), the intentional phase follows when interest in the study has been sufficiently formed [35, 39]. For the topic of study participation, this means, that the focus is on questions such as how participation can be realized given certain barriers and support factors, whether difficulties can be adequately addressed, if sufficient resources are available (e.g. equipment for online studies), and what support is available.

When participation is considered feasible, the participant enters the actional phase. Participation experience can be evaluated by repeatedly comparing current attitude to original participation intention and expectation. When intrinsic (especially knowledge gain) and extrinsic interests and goals (including meaningful support of research, appropriateness of effort and incentives) are met, commitment and motivation should remain stable. If not, the probability of a drop-out increases. The final, summative evaluation of study participation is also important for successful recruitment, since it determines participants’ willingness to participate in follow-up studies or to recommend participation to others.

**Fig. 1:**
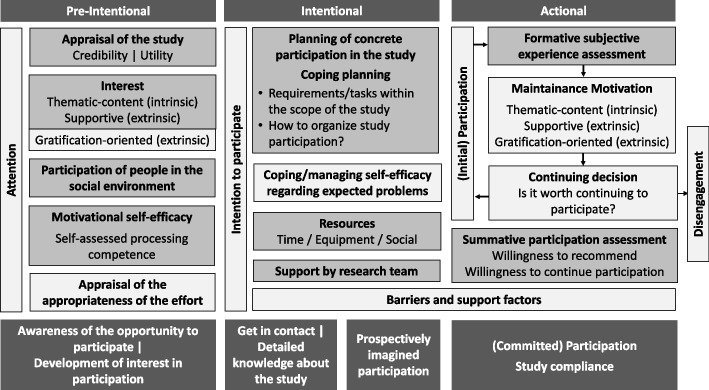
Framework of intentional and actional components of engaged participation in public health research studies (INTACT-RS)

Figure 1 shows the components introduced above, based on the Health Action Process Approach (HAPA, [39]), which describes health-related behavior change processes. When transferred to the topic of research participation, it is important to consider that the opportunity to participate occurs externally and that the fit between a person’s interests and study content is considered fundamental. Lastly, while HAPA aims at long term health behaviour promotion, in research recruitment the actional phase already implies that engaged participation was achieved.

### Rationale and objectives

In the multicentre research network “Health Literacy in Early Childhood Allergy Prevention” (HELICAP, FOR 2959), six research projects investigate the role and concept of health literacy in the context of early childhood allergy prevention (ECAP) and COVID-19 infection prevention (COVID-19-IP). The main target groups, i.e. parents of infants, pediatricians and midwives resemble the diverse challenges related to recruitment, not least in terms of creating interest, a lack of time of participants, and identifying effective incentives; the COVID-19 pandemic further reduced the usual opportunities for communication and contact. Certainly, these groups also offer opportunities for approaching them for study participation, particularly the common interest in child health. These conditions required a strategic, theory-focused approach to recruitment, by means of which starting points and methods could be justified, and from which extensive empirical evidence could be gained. We aimed to answer the following questions:

1. How is each framework component defined, characterized and concretized by experiences from the case studies?
2. Which pre-intentional, intentional and actional participation considerations do potential study participants face in each phase of decision-making and participation?
3. How can these considerations be accounted for when planning the recruitment process according to the framework (action guidance)?

By answering these primary questions, we also aim to better understand which components and processes within our framework are especially decisive for an individual’s decision to participate and remain in a research study, and whether its application has (positive) implications for the validity of collected data.

## Methods

The research network (HELICAP) comprises six independent research studies, four of which involved recruitment activities (see Table 1 for details). Study 1 examined how pediatricians and midwives consider parental health literacy during their counselling on allergy prevention. Study 2 and study 3 investigated parental health literacy, by analyzing knowledge, (information) practices and needs regarding allergy prevention. Due to the pandemic, both added Covid-19 infection prevention as a second use case. Study 4 related to the accompanying research done by the research networks’ coordination centre, aiming to establish a “parent board” to facilitate patient and public involvement. Overall, while each recruitment entailed multiple formats and target groups, we relied on similar recruitment principles according to the framework described below, and aimed to create organizational synergies, for instance by applying a common contact management database.

**Table 1:** Summary of studies

Study	Use case	Purpose	Study design	Sample	Recruitment	Gratification	Available material
1	Early childhood allergy prevention	To explore how German health professionals in ambulatory care take up, make sense of and transfer research evidence on ECAP to their patients’ families and how they consider HL challenges in their ECAP counselling and organization of their practice setting	Qualitative: semi-structured individual telephone interviews	Health professionals (*N* = 43) • midwives (*n* = 24) • pediatrics (*n* = 19)	• multipliers (professional associations) • cold calling • snowballing	€50	• Email responses by participants and recruitment partners • Notes of conversations before and after the actual interview • Notes from telephone calls and personal conversations
2	a) Early childhood allergy prevention	To explore parents of infants’ awareness, relevance, knowledge, information behaviour, information appraisal and information preferences	Qualitative: semi-structured digital and non-digital focus groups; individual telephone interviews Descriptive: health literacy survey, socio-demographic data	Parents (*N* = 114) • Parents-to-be, or • with infant children • with and without allergy history	• multipliers (professional healthcare, social, and patient organisations, pediatrics, midwives, allergists, childcare) • digital and non-digital approach • local and (supra)regional contacts • individual, face-to-face recruitment (e.g. playgrounds) • snowballing	€30 / €50	• Email responses by participants and recruitment partners • Notes of conversations before and after the actual interview and focus group • Individual tracing of each participant’s way into the study
	b) COVID-19 infection prevention	To explore parents of infants’ information behaviour, trust-based behaviour and information needs and preferences		Parents of infant children (*N* = 30): • Arabic (*n* = 10) • German (*n* = 20)		€30	
3	Early childhood allergy prevention; COVID-19 infection prevention	To develop and validate a measurement instrument for parental health literacy	Quantitative: 3-staged online questionnaire (45 min each)	Parents (*N* = 492) • Parents-to-be, or • with infant children • with and without allergy history	• multipliers (health insurance, healthcare staff, pediatrics, midwifes, kindergarten, clearing offices) • digital and non-digital approach • visual (flyers, posters) & audible (radio contribution) • individual/private (individual contacts) and public (e.g. playgrounds) • local and (supra-)regional contacts • snowballing	• €30 • lay summary of ECAP evidence • expert lecture and Q&A event	• Email responses from participants and recruitment partners • Notes from Telephone calls and personal conversations with participants • Quantitative data on recruitment pathway, allergy status, and COVID-19 infection status, summative assessment of study participation
4	Early childhood allergy prevention	To strengthen patient and public involvement in the process of conducting research for specific target groups	Qualitative: participatory study, face-to-face groups	Parents (*N* = 14) • Parents-to-be, or • with infant children • with allergy history	• multipliers (patient organization, nursery schools, kindergarten) • private, individual contacts • previous studies	• €30 • Compensation for travel expenses	• Email responses from participants • Notes from personal conversations with participants before and after the actual focus group

In a first step, we developed the conceptual framework: We reviewed existing psychological models of health behaviour that explain the development of intentions and the planning and implementation of behaviors [40]. The social cognitive process model of health behavior [39] – confirmed many times subsequently – includes a health behavior theory that differentiates pre-intentional motivation (intention formation) and post-intentional volition (initiation, maintenance) [41]. This conceptualization relates to the topic of recruitment, as initial non-willingness needs to be changed to successful participation, and hence HAPA is a suitable theoretical basis for participation behaviour. Since more recent literature focuses directly on facilitators of study participation, we further specified the framework with respective constructs; i.e. we reviewed (recruitment) literature that includes conceptual approaches to (potential) participants’ consideration and appraisal processes [5, 7, 8, 27, 42, 43] and included these elements into the framework. For instance, the phase of intention formation relates to aspects of attention, credibility, extrinsic and intrinsic interest, and appraisal processes being determined by interaction with the social environment.

In a second step, to further define and inform the theoretical framework components with empirical evidence, we followed basic principles of thematic- and qualitative content analysis (QCA) [44–46]. QCA is an established method for analysing qualitative data – here, participant responses – as it allows for structuring and assigning text-based data inductively or deductively and to identify patterns in the data. First, female and male researchers from all studies (Master or PhD degree in Public Health; JL, AH, JvS, AAS, CD, HA, GS) compiled all recruitment-relevant responses from study participants (study 1-4, Table 1), including the sources and (researcher’s) notes that documented the study and recruitment planning (step 1: initial text preparation). Also, we prepared the table of 15 framework components to serve as deductively-derived main categories (step 2: category building).

Then, two researchers assigned available material – mostly single sentences built the unit of analysis, more sentences were added when further understanding was required – from each study reported here to the 15 components (Supplement 1). To do so, we identified (coded) a keyword or key-phrase in each quote/quote passage, using a pre-defined data extraction template in Microsoft Word. Based on the keyword/code, we preliminarily assigned each quote to the most suitable framework component and resolved remaining and unclear quotes by discussion with three further researchers (step 3: coding). As a result of the assigned text material, we also identified main- and sub-components of the framework (see below).

Next, according to the category-based overview of the material, we drafted broad summaries of each framework component according to a) participants’ behaviour during recruitment, and b) actions taken by the recruiters. We then identified similarities and differences for each recruitment process (studies 1-4) and, based on that, iteratively refined and complemented each components’ description (step 4: analysis – component-related thematic summary). The analysis was carried out in accordance with the criteria for reporting qualitative research provided by Tong et al. [47].

## Results

The results section includes all framework components highlighted in grey, for which data from at least three studies described here could be applied. All other model components are summarized in Table 2, with a full description in Supplement 2.

**Table 2:** Framework sub-components

Component	Description	Example quote
Phase: Pre-intention		
Attention	The distinct and resource-dependent channels to generate initial attention of the target group via distinct access levels (individual, organisation, public) and access contents (e.g. study topic vs. study process).	“I read your ad on Facebook and am very interested. I also meet all the criteria. If you are still looking for participants, I would be very happy to hear from you!” (S9)
Interest: Gratification-oriented	Financial interest proved to be important especially for people with lower socioeconomic status. Elsewere content and support oriented interests were decisive.	“Unexpectedly, I learned a lot (…). So I will be able to take away more from this survey than the allowance. Thank you very much!” (S72)
Appraisal of the appropriateness of the effort	The eventual judgment potential participants make to decide whether to join the study, which depends particularly on the relation, i.e. balance between required effort (e.g. time, organizing childcare) and expected outcome, i.e. personal benefit.	“As I come from XX, attending for only 3 h would be too time-consuming for me (…). The cost-benefit factor does not make sense for me. Perhaps digital participation would be possible if you made it a hybrid event.” (S111)
Phase: Intention		
Coping / managing self-efficacy	The internal consideration process in which participants estimate, appraise their own abilities for successful participation and whether strategies (skills) are available to handle expected or unexpected problems.	“I would very much like to participate in your project. However, I am still fully breastfeeding and am not sure if it is feasible with the baby in October (from XX district). Would the baby be able to be at the meeting? Otherwise I would be very happy to be there outside the meeting.” (S141)
Phase: Action		
Maintenance motivation	Three foci: thematic-content, supportive and gratification-oriented. Maintenance motivation is more pronounced in the actional phase, as it involves actual participation experiences at this point.	“Basically, after the first interview I already found it difficult to motivate myself for the next one. The feeling became stronger during the second interview. The connection to the topic was missing, I didn’t see any sense (…).” (S217)
Continuing decision	A participants’ decision to continue with follow-up assessments after initial evaluation considerations. Reasons for early dropout vary, but often relate to time constraints.	“I’ll ask my husband again (…), but due to the shortage of time at the moment - I’m guessing he won’t be able to make it.” (S227)
Phase: Overarching		
Barriers and support factors	The specific factors that narrow down an individuals’ chances to decide in favor of study participation, and that differ according to each framework phase, e.g. missing technological resources during concrete participation planning (intentional phase) or a lack of time to participate in subsequent phases/assessments as part of the initial study (actional phase), which require development of action steps to create ‘support factors’, e.g. strong content-wise links of the study with the interests of (potential) participants.	–

### Pre-intention phase

#### Appraisal of the study

After an individual’s attention has been directed to a study, they move on to the appraisal phase. Firstly, potential participants seek to determine a study’s credibility, for which they consider visual and written cues, particularly in terms of the host institution (publicly funded research organization) and its status (name, logo, popularity) as well as information about the people conducting the study (S17-S22). Credibility was also conferred by the cooperation of entities already present in the lifeworld(s) of the target group; here, midwives, insurances companies, childcare facilities, umbrella organizations (for HCPs), and patient organizations: *“It helps me a lot when someone else I trust recommends participation. Then I don’t need to waste time on finding out about its trustworthiness”* (S23). Partner organizations’ connections to the target groups’ lifeworld seemed to confer greater credibility than unfamiliar independent scientific institutions. Further, credibility appeared to be influenced by the information about potential gratification, e.g.: *“Where does the money come from and is it really paid out?”* (S20) and *“Is there a catch, am I now committing myself to something?”* (S21). On the other hand, financial compensation may reinforce credibility, as demonstrating funding by an official authority.

Second, study calls were appraised in terms of utility, i.e. the benefits from participation (S24-S26). Research topics such as health literacy or health information are not per se evaluated as *“I should support this research because it will enable significant scientific advancements”* – except for those intrinsically motivated to support health research (S26). Interested individuals may rather consider utility as direct personal benefits, which we aimed to emphasize by pointing at the topics’ relevance from the perspective of parents (e.g. importance of effectively using health information to make child health-related decisions) and HCPs (e.g. enabling patients to make informed health decisions), and needs for improvements (developing evidence-based ECAP information), which then benefit the target group, e.g. in effectively preventing allergies (S24, S27, S38).

Overall, it was vital to consider the different meanings of utility from the participant’s perspective in advance. For instance, later conversations with parents (study 2) showed that learning from others’ experiences may also be judged to have utility (S28-S31). However, it was difficult to distinguish utility judgments from “interest” (below), e.g. written and electronic registrations revealed mainly intrinsic and/or extrinsic interests, which indirectly relate to personal or social benefits.

#### Interest

##### Thematic content

Depending on whether interested individuals can relate to the study topic (e.g. high concern about allergic predispositions, professional activity), expectations may differ and misconceptions can arise. Especially for people with a strong thematic interest (“*Our daughter is 2 years old and suffers from multiple allergies. Therefore, the topic plays a big role in our everyday life.”* (S32)), the subjectively-perceived study goal may differ from the realistic study goal. In our case, interested parents aligned ECAP with their individual situation, for instance: *“I would like to register for the hay fever study”* (S33), *“registration for the asthma study”* (S34). In the short term, this increases motivation to participate, but could also cause frustration if an expected individual benefit falls short. To increase thematic interest while informing participants realistically, we stated clearly both the study objective and participation benefits. As prevention of disease in infants usually has a high subjective value for any parent, but ECAP may rather concern those with a predisposition (S32, S35-S51), we emphasized not only ECAP but also child health in promotional materials and invitation letters. To recruit HCPs, ECAP was relevant as an overall (child health) and a specific subject, as it is a part of routine counselling [48].

##### Supportive

For those individuals whose intention formation strongly relates to solidarity, we identified three major reasons for support. Firstly, participants may want to reciprocate the value of research with an equivalent action (Reciprocal action: *“wanting to give something back”,* S56; S57-S61)). Secondly, participants may want to advance research (support for science: *“My older daughter suffers from allergies, and it is very important to me to support you in this study […] In my opinion, little information is available on the prevention and management of allergies in children”* (S62; S63, S64)). Thirdly, individuals may want to support those in similar situations (solidarity with peers: “*In my family, there are strong allergy sufferers. That’s why I’m keen on the success of your study*” (S65; S66)). Particularly for topics characterized by insufficient or rapidly changing evidence, potential participants are more likely to participate once they expect a knowledge gain. Hence, we applied the principles of priming (e.g. visual priming by providing a portrait of the contact persons), social reference (e.g. recruitment partners referring their members to the study), and emphasized the benefits for society (macro level, e.g. insights into HCPs’ communication with parents will help inform allergy education).

#### Participation of people in the social environment

For each recruitment process, we observed that the social environment of study participants is key during intention formation. For instance, a considerable part of expectant and new parents included in study 2 reported frequent engagement with family, friends, and parents in similar phases, for instance to exchange on daily childcare routines (S1, S78-S82, S100). HCPs understood the social environment as contacts to colleagues, to some extent, research institutions, who referred them to the study as part of their professional communications (S83, S84).

Obviously, the social environment creates an opportunity to get attention, e.g. when previous participants share the invitation with their immediate contacts (snowballing) (S79, S81, S83-S88). In study 3, about half of the study participants became aware of the study via friends and family members. This effect was particularly relevant for fathers, as they were usually harder to reach. In study 2a/b, already-included participants repeatedly mentioned having circulated the call via instant messaging and social media (S85). Besides attention, the social environment may also help to appraise participation credibility, utility and required effort, as individuals seek guidance in what other people deem relevant (S85) [49]. If one’s own social circle report positive, valuable experiences, then those who are in a consideration process are more likely to participate, too, based on socially-proven patterns (S82) [42]. Further, we found that the social environment could affect individuals’ motivation: particularly in study 1 with HCPs, higher degrees of attachment were observed once colleagues and umbrella organizations encouraged participation (S83, S84, S89, S90).

#### Motivational self-efficacy

“Motivational self-efficacy” here refers to potential study participants appraising whether they can effectively participate in face-to-face communication, which is different from, e.g., participating in an individual interview, which could be observed primarily in study 2a/b. As various interested participants and recruitment partners expressed concerns about group discussion participation not only in terms of time or technical requirements, but also due to doubts about communicating within a group, interested participants may implicitly or explicitly appraise their communicative abilities when the study format requires group interaction (S100-S103). We therefore added the option of individual interviews. Besides, motivational self-efficacy also referred to parents’ confidence in their ability to answer ‘unknown’ research questions as an expert in their own living environment and benefit the research study (study 2a/b, study 3). Parental self-efficacy is particularly obvious in families where allergies were not yet present and where uncertainties arose about their suitability for study participation *(“May I still participate if I don’t know anything about allergies and no one in my family suffers from allergies?”* (S104; S105, S106). Transparently communicating the study’s objective and researchers’ expectations, assists parents in appraising and motivating their participation. Hence, intention formation is not only impacted in terms of “what needs to be done to promote the study?” (e.g. awareness) but also regarding “how do potential participants judge their own abilities?” While motivational self-efficacy did not play any role for recruiting pediatricians, it was relevant for midwives (study 1), who sometimes voiced doubts about their ability to provide relevant insights (S107).

### Intention formation phase

#### Planning of concrete participation in the study (coping planning)

Here, a first aspect concerns specific questions regarding the study’s contents and format, e.g. study scope, mode of questioning (questionnaire, interview, focus group), format (e.g. personal (live) or impersonal (survey)), technical requirements, time and duration. Participants consider whether respective requirements are in accordance with personal resources and capabilities (S102, S114-S116). When planning recruitment, we aimed at specifying any relevant aspect as part of the study call, so that interested individuals could make use of sufficiently detailed and complete information, also to enable a step-by-step appraisal of the requirements. Further, to reduce participants’ doubts about, for example, being able to answer survey questions (S117-S120), we reframed initial, rather abstract research objectives (e.g. understanding parents’ ability to seek and apply health information) in such a way that they would more closely resemble situations encountered by the target group(s) as part of their private or professional life, e.g., receiving information and/or recommendations specific to child health (parents), or providing advice about how and where to find health information (HCPs). As such, the study calls also included summaries of actual study questions, so that participants could imagine the actual study situation.

#### Participants’ resources

Besides general and content-specific expectations, interested participants relate their participation to available organizational and technical resources. Firstly, time appeared critical for parents and HCPs, particularly during the day, when other time-consuming tasks take precedence (S107, S149-S153). These needed to be anticipated and often required flexibility, e.g. parents of infants needing to pause survey completion (study 3). Regarding (live) participation in online focus groups (study 2), the point in time and the duration appeared critical, which equally applied to in-person meetings. Our target groups often considered a 60–90 min evening online appointment acceptable, whereas longer in-person meetings often were not (S154-S156). HCPs are constrained by opening hours, which repeatedly required flexible appointment alternatives, e.g. during lunch breaks or after working hours.

Further, we found resource judgements related to concerns about technical equipment. For individual interviews (study 1, study 2a), participants welcomed easy access options (i.e. telephone interviews) to reduce technical requirements and increase flexibility *(“Can we conduct the interview right now?”* (S157); *“Can I call you once I’m finished with my patients?”* (S158)). In contrast, online focus groups and interviews (study 2) and online surveys (study 3) entailed multiple considerations related for instance to connection stability, use of technology (camera, microphone), and handling of distractions during participation (S118, S159). While not all technical hurdles may be anticipated, we aimed at accommodating resource considerations by providing concrete advice and offering personal assistance. The estimation of required resources and whether sufficient support will be available during the study appeared particularly relevant for hard-to-reach individuals such as non-native speakers. Here, language assistance during interviews and focus groups (study 2) needed to be guaranteed before respective individuals agreed to participate (S100, S120, S160, S161). Depending on the target group and study format, resources and support may also relate to additional aspects, e.g. to provide facilities to accommodate infants’ needs, such as the possibility to breast-feed or offering childcare during interviews (S102, S103, S141).

#### Support by research team

Apart from considering the perspective of potential participants in terms of (their) resources, intention formation and participation planning benefited from a range of support measures, which, in our cases, included for example individual negotiation of (additional) timeslots (S169, S170), help with survey completion (S171), and providing feedback about measures for data protection (S17, S18). Though these may generally be handled by standardised procedures such as written summaries, participants with specific concerns appreciated personal feedback (S24, S174- S176). While this is time-consuming for researchers, having an opportunity of direct communication (study 2: offering instant messaging) resulted in creating relationships of trust, which reduces drop-out and no-show rates.

### Actional phase

#### Formative subjective experience assessment

Once participants have become part of a study, initial as well as any further participation is formatively evaluated either during participation or subsequent reflection processes. In study 2a (interviews), to gain explicit insights into participants’ subjective assessments, parents were asked for their evaluation towards the end of the interview. Positive experience criteria included the comprehensibility of the questions, the entertaining nature, the credibility of the discussion and the benefits in terms of content, which, for instance, also included to inform their own health information seeking behaviour (S191-S193). In study 3, participants reported uncertainties arising from whether they answered the (multiple choice) questions correctly (S194-S197). On request, sample solutions were forwarded to participants, so they could turn uncertainties into secure knowledge. These positive experiences increased motivation and prevented early dropouts. This study also revealed that formative evaluation significantly influences the quality of the data; when it is weak, study questions and tasks are addressed with less care. Hence the motivation to continue participation primarily resulted from the formative evaluation (study 3), as other factors such as social control and desirability were less relevant due to anonymity and online questioning.

#### Summative participation assessment

When reflecting on the process of participation, participants determine overall satisfaction (S72, S234-S245) their willingness to join other future studies (S71, S246, S247) and whether they would recommend the study to peers (S88, S97, S99). Given the relevance for the study’s continuity and sustainability, these (personal) aspects should be included in a formative and/or summative project evaluation — to draw conclusions for further projects. For example, a summative evaluation may include questions about the willingness to recommend the study to others (study 3: 89%, *N* = 438/492), adequate individual use of resources, whether the study was interesting overall (study 3: 94,9% (*N* = 467), and whether financial compensation is appreciated (study 3 (90,4%, *N* = 445). A completely or almost completely positive summative evaluation repeatedly encouraged participants to recommend participation within their near social environment (study 2, study 3), boosted new participants’ initial appraisal (pre-intention) and helped to reduce hesitancy.

## Discussion

The analysis of the target groups’ participation considerations revealed indicators for all components of the INTACT-RS framework. This provides exemplary information about motivational and actional processes, which are decisive for committed research participation from the perspective of participants. While parents and HCPs may not be perceived as “hard to reach” groups per se, we expected recruitment to be challenging [9, 23, 26] and aimed to understand this process.

The literature mostly addresses recruitment barriers and facilitators [25, 29, 50]. Regarding barriers, those that are repeatedly mentioned across studies relate to (insufficient) recruitment training, including communication skills vis-à-vis study participants, the complexity, length, and availability of study information, available recruitment resources, concerns within the target group about why and how to participate, cultural and/or language-related factors that hinder participation, and the availability and appropriateness of incentives [9, 15, 17–19, 24, 28, 50]. These barriers are often grouped according to different levels for which barriers can occur, i.e. study-related, design-related, recruiter-related, and participant-related [15, 18, 24]. Our article instead focuses on overcoming obstacles to successful recruitment by setting starting points for action (supplement 3). Also, even though we identified barriers and facilitators in the pre-intentional phase, it can be assumed that they predict volitional abilities that only appear after intention formation [35, 39]. This is because barriers and facilitators determine when and under what conditions an action is initiated and formed. Hence, and according to the original HAPA model [39], this framework component may rather need to be located in the intentional and actional phase.

Besides factors such as recruitment channels, methods, and necessary prerequisites, INTACT-RS shows that there is a different way of understanding what can hinder an individual’s participation when going into more detail, e.g. insufficient attention to the opportunity to participate in research or a negative perception of one’s own ability to participate successfully. Hence, emphasizing psychological and behavioural aspects of ability, coping, motivation, interaction, decision making and action control can be important; in the context of citizen science, Lotfian et al. [43] mapped reasons that would motivate participation by individuals into a framework of intrinsic and extrinsic factors, which relate to the interest components (thematic, supportive, gratification-oriented) of our framework in a similar way. However, with our own findings we hope to demonstrate that motivation is (only) one, though important, part of a more holistic approach to better integrate psychological and behavioural aspects in the planning and conduct of research.

The analysis of barriers also typically centres the (ongoing) recruitment process, e.g. in terms of information material or communication with potential participants. However, when applying INTACT-RS, it became clear that participants’ appraisal and consideration may already happen prior to engaging in communication with the research team about how and when participation is possible, and a final decision e.g. about continuing in a follow-up is made with some (temporal) distance.

An even greater proportion of existing studies recommends recruitment ‘facilitators’. These often relate to retrospective descriptions of what researchers and recruiters considered helpful, i.e. experience-based recommendations [19]. While various facilitators have been identified for specific study settings and objectives, such as recruitment during unscheduled hospital admissions [29], reviews reveal various generic facilitators: cooperating with a HCP to approach target groups (doctors for clinical trials), directly and personally approaching potential participants, strengthening recruitment skills of those who do the recruiting, sufficient allocation of resources for recruitment, and selecting a specifically suitable recruitment site. Research has also identified participant-related reasons for joining research studies, particularly the desire to help others, face-to-face interaction with recruiters, and receiving a recommendation from peers. However, such rationales are mostly inferred from researchers’ observations and experience, and without taking a more structured, inclusive perspective of the spectrum of (all) potential determinants.

In our research, we related potential recruitment facilitators to each component within INTACT-RS to enhance understanding of the perspective of (potential) study participants. In fact, there have been valuable previous efforts to incorporate participants’ perspectives about recruitment in a framework, for instance for “Rationale for research participation framework” by Weller et al. [51]. While this specific framework example entails reasons for participation, e.g. participation to help others, we focus on the steps and phases potential participants go through, so that researchers can directly align their respective measures and steps for recruitment and study conduct to each component. In that sense, using empirical insights about what facilitated recruitment and retention, we formulated a total of 41 recommendations alongside the framework components, i.e. considerations that may be taken up by researchers (Table 3). The component ‘barriers and support factors’ is considered separately from this checklist in supplement 3.

**Table 3:** Checklist: guiding questions from the participant’s perspective and recommendations based on INTACT-RS

**Preliminary considerations**		
a. Situation/condition analysis to identify target group-specific interests and needs, behaviors and requirements that arise for recruitment regarding the target group-specific situation b. Stakeholder analysis to identify relevant multipliers in the target group & living environment c. Resource plan for active communication structures to maintain contact throughout the participation process (e.g., individual contact persons, regular contact and inquiry about problems and challenges)		
**Phase**	**Questions considered by potential participants**	**Actions researchers could take**
**Pre-intentional**		
1. Attention: Taking notice of a study	Does the study call address me? Does it attract my attention?	Facilitate getting attention by describing/highlighting a question, topic, or concern that is (currently) relevant in the target group’s daily life (rather than only describing a generic study objective)
		Considering different ways of approaching the target group given the available resources (e.g. time, staff, money)
2. Appraisal: Determining the study’s credibility & utility	What is my first impression of the study? How do I know the investigation is credible? What is in for me?	Allow potential participants to get a good idea of the people who conduct the study and visualize/personalize the host institution for instance by providing potential participants with clear information about the people conducting the study and portraying the research team
		Transparency in the description of the study process and the study objective (incl. data protection, funding sources)
		Description of the relevance to the target group (incl. realistic harm and benefit)
		Considering the influence of cooperation partners that are present in the lifeworld(s) of the target group on the credibility judgment
		Establishment of a participant-researcher relationship by transparent and active communication
3a. Interest: Participating because of the study’s topic	What would be the reasons for me to participate? Will the study answer questions I have?	Review the research question and topic before the start of recruitment in view of the target groups’ interest and motivation
		If necessary, reframe, i.e. ‘translate’ research question and topic from the researchers’ to the participants’ perspective (plain language)
		Cite experiential reports from targeted participants that potential participants can identify with
3b. Interest: Participating to help others	Could my participation be of help for others?	Provide a realistic argument in the study call for whether and how participation will benefit others and if so, when this help will take place
		Give specific examples that demonstrate how the study & findings can help
3c. Interest: Participating for a (non-) material incentive	What will I get back for participation?	Adapt a material/financial/ personnel incentive according to target groups preferences/needs: a sufficiently high remuneration for target groups who may prefer money (e.g. €50 for a 1-h duration), donation of material or financial help to a social, health, or environmental fund, the provision of personal competences (e.g. through an event or the supply of specific information), which benefit the participants in their daily lives, or let participants decide about the preferred type of incentive
4. Social environment: Being guided by others	Have I heard back about the study from others around me? What do they think?	Identify whether and which non-professional individuals act as reference persons to the target group, e.g. social media bloggers, patient advocates, and include them early on in the recruitment
		Identify individuals with a particular (thematic, intrinsic) motivation for participation and involve them actively as peer recruiters – provided their role and benefit is clear
5. Self-efficacy: Having confidence in oneself	Am I confident about successful participation? Would I be able to handle the demands?	Clear description of the study requirements
		Ensure an accessible study centre or an individual contact person who can immediately clarify questions and provide individual solutions in case of difficulties
		Provide examples of situations and/or task, requirements, and skills the target group is already familiar with, that will feel them to participate successfully
6. Appropriateness: Considering whether participation is worth it	What will I put in to and get out of the process? Would I rather invest or get back? All things considered: Will the effort be worth it?	Pretesting of the individual effort by the target group
		Derive and list potential barriers and facilitators that participants may encounter and revise requirements if barriers outweigh facilitators
		Engage institutions and individuals in the recruitment process that are in regular and trustful contact with the target group and can hence provide orientation for potential participants about the pros and cons of participation
**Intentional**		
7. Planning: Preparing for participation	What exactly do I have to do and is preparation necessary for that?	Establish a pre-participation conversation (mode) with participants to guide them through the planning (accessible accompanying communication structures)
8. Coping: Handling expected challenges	Could there be any problems during participation? What can I do in that case?	Active communication to strengthen trust in the participant-researcher-relationship and own capability
		Instructions in plain language concerning technical requirements
		Include examples in the study call and/ or pre-participation communication that mirror the study (situation and-or) content closely
9. Resources: Arranging for participation	Do time, technology and organisation play a role for participation? If so, how do I arrange for it?	Provide organizational, technical and scheduling alternatives for individuals in particular who express respective doubts to ease planning
10. Support: Relying on help	Is there any available support before and during participation?	Guarantee the availability of a low-threshold and (short-term) support throughout preparing and taking part in the study
		Preparation of an overview for participants “in case of…”
		Use of familiar communication tools of the target group (e.g., instant messaging, e-mail) to improve direct communication
**Actional**		
11. Formative Experience: Giving (initial) feedback	What is my (first) impression of participation? Were my expectations met?	Enable concrete feedback mechanisms, e.g. formal or informal conversation or survey at the end of initial participation to be able to adapt the recruitment process
		For multi-part/longitudinal studies: Establish regular monitoring structures to capture difficulties and uncertainties of participants during the participation process
		Incorporate initial feedback from early included participants into ongoing recruitment and-or study conduct if possible, for instance to use previously ignored recruitment channels or adapt the study call
12. Motivation: Motivating to maintain and continuing to show interest	Does the study still catch my interest? Am I still motivated? Is the gratification worth continuing?	Use reminder features to emphasize personal benefit and both addressee and societal relevance, and to remind people of the gratification
13. Continuing: Deciding about what to do next	Do I want to continue with the steps and tasks that are still to come? Is it worthwhile to continue participating and do I actually continue?	Strengthen participants’ feeling of attachment to the study by giving them an active role (see above) and provide a clear plan for the following steps
		Focus on timely commitment - try to reduce time needed for completion for follow up studies
		Point in time for follow up studies should be communicated clearly
		Contact regularly to inquire about difficulties and problems during the participation process
		Provision of tools to assess reasons for early drop-out (drop-out analysis)
14. Summative assessment: Completing the study	My overall judgment? Would I recommend the study to others?	Scheduling a final survey for summative evaluation of study recruitment as a learning process for future recruitment
		Appreciative final thanks: Personal feedback that highlights the relevance of participants contribution
		Promoting the perception of the credibility and relevance of scientific studies in general
15. Barriers and facilitators	What stops me from participating? What strengthens my participation?	Addressed in supplement 3

Looking at pre-intention, we could relate this phase to some of the previously established facilitators, particularly the help of recruitment partners such as health- and social care facilities that are in close contact to target groups [17], or the benefits from direct, personal contact to desired participants [52]. However, firstly, public health research often relates to topics that are not inherently medical, which may be out of scope of those working in a healthcare practice. Secondly, (potential) participants strongly welcome feedback from institutions they trust [20] for instance to appraise credibility and utility. Nevertheless, we realised that, due to workload, e.g. pediatric practices can provide little assistance despite their proximity to parents, while (medical) umbrella associations were rather helpful to approach HCPs. Therefore, and somewhat in contrast to other ‘classic’ means to increase attention, such as the allocation of resources [15] and training [28], our own efforts focused on directly appealing to an individuals’ awareness, motivation, interest – for instance by relating the study’s topic more closely to current interests, questions and possible concerns prevalent among the (distinct) target groups.

As outlined above, (previous) recruitment research first and foremost addresses aspects related to recruitment planning and ongoing recruitment as such, including but not limited to the identification of effective recruitment channels, recruitment staff, methods for approaching individuals, and the design of recruitment information materials. While these factors relate to the crucial phase of raising attention, INTACT-RS attempts to enhance this step via the phases of intention formation and action. Regarding the former, we identified various aspects (framework components) that can no longer be ascribed to initial attention and awareness, but that only evolve when individuals plan for participation in more detail, e.g. handling of technical and organizational resources. The examples of facilitating an individual’s planning by providing participation alternatives (e.g. mode of participation) when necessary and reducing doubts about being able to participate successfully through personal and direct assistance, illustrate that individuals relate their successful participation in research to aspects beyond initial interest and that cannot be covered only by the allocation of recruitment resources, the support from recruitment partners, etc.

Regarding the latter – the actional phase – while participation already takes place at this stage, recruitment and retention are still relevant, particularly because of participants’ subsequent decisions: to continue with the study, for instance in subsequent data collection or follow ups, participants reconsider interest and motivation, i.e. compare expectations with actual participation. To recommend (or not) participation to others, participants require a clear understanding of the importance of this task for the success of the study and hence, specific advice on how they can contribute to this. Given the continuing discussions about effective recruitment strategies (e.g. [9, 25, 53]), it may be legitimate to integrate these and related aspects (see Table 2) into study planning, rather than (only) focusing on ‘how’, with what means to approach potential participants (e.g. [18, 26]). Previous work by Nov et al. [54] highlights how (initial) motivations, interest, etc. impact on the (subsequent) quantity and quality of participation, providing another argument for why it is important to use a more comprehensive framework.

INTACT-RS supports the validity of the collected data in addition to the general willingness to participate. In particular, the validity of questionnaire data that assess subjective judgment and perception is promoted by motivated and serious study participation. Response biases such as halo effects, self-serving biases and sequential effects occur especially when participants tend to answer in an unfocused, facile manner and in the mode of impression management [55]. Also in qualitative focus groups, the interest, motivation, and mutual perception of the communication partners moderates the validity, depth, and sincerity of the individual responses [56]. The better the process elements explicated in INTACT-RS are accounted for, the more likely the specific item contents may be processed in depth and seriously considered.

While INTACT-RS relies on a the previously established HAPA model [39], its further application will reveal new insights and its individual components, though justified by empirical evidence, may not be exhaustive. Based on the available data, some components could be characterized in more detail (e.g., interest) than others (e.g., maintenance motivation). Hence, QCA is useful to indicate an overview/spectrum of topics – here, recruitment challenges – but cannot fully validate a framework, also it implies some degree of giving meaning to participants’ statements as part of the coding process. Further empirical testing is needed to clarify which framework components are a) valid main indicators of active participation and b) predictors of a valid data situation. In addition, it still needs to be determined whether the components’ relevance differs depending on the target group. A structured assessment of the perspective of how study participants – and possibly researchers perceive each component, may induce changes to the framework. Since we advocate here for strengthening participants’ perspective on recruitment and retention, active involvement into the frameworks’ further development seems indispensable. This would also be important since the studies underlying the recruitment processes assessed parental information and prevention behaviour, not study participation intentions, decisions, etc. and could hence be interpreted as ‘secondary’ data. While for instance an assessment of non-response reasons – which this study does not entail – would further objectify the reasons for or against participation, even the responses gained from this research reveal the many hurdles of attracting (potential) participants’ attention. These desiderata constitute the prerequisite for identifying interventions that promote the motivation- and interest-related processes postulated here.

## Conclusion

Though it is (potential) research participants who eventually agree to or decline study participation, previous research often emphasizes what research and recruiters can do to facilitate recruitment. Also, taking part in research does not only relate to successful recruitment, but entails phases before and afterwards. Therefore, INTACT-RS aims to increase the integration of basic behavioural and motivational components which inform an individual’s decision-making, to enable a systematic recruitment and retention approach.

## Supplementary Information

**Additional file 1.** Excerpt of the qualitative data material and relevant anchor citations.

**Additional file 2.** Full description of further framework components.

**Additional file 3.** Identified barriers based on the INTACT-RS framework.

## Data Availability

The qualitative (interviews, focus groups) and quantitative (survey) data underlying the initial studies reported here can be accessed upon request from the corresponding author.

## Abbreviations

HCP: Healthcare professional
HAPA: Health action process approach
ECAP: Early childhood allergy prevention
COVID-19-IP: Covid-19 infection prevention
INTACT-RS: Intentional and actional components of engaged participation in public health research studies
