# Supplementary material for: Intentional and actional components of engaged participation in public health research studies: qualitative synthesis of a recruitment and retention process into the theory-informed INTACT-RS framework
Source: BMC Med Res Methodol. 2023 Jan 16;23:17. doi: 10.1186/s12874-023-01838-3 (PMC9841138; doi:10.1186/s12874-023-01838-3)
Supplement: Supplementary file 1 — Additional file 1. Excerpt of the qualitative data material and relevant anchor citations. [file 12874_2023_1838_MOESM1_ESM.docx]

**Supplement 1.** Excerpt of the qualitative data material and relevant anchor citations

**Pre-intention**

**1. Attention**

1. „Eine liebe Freundin hat uns über Ihre Befragung Aufmerksam gemacht. Ich dachte ich melde mich bei Ihnen einfach mal, mein Mann und ich würden gerne teilnehmen.“ (W6M-DI01)
   Translation: "A dear friend of mine told us about your survey. I thought I'd just get in touch with you, my husband and I would like to take part."
2. „Guten Tag, ich habe ihren Flyer zugesendet bekommen wegen den Allergien würde da gerne mit machen meine Tochter ist 5 Wochen alt und ich bin Allergiker daher interessiert es mich sehr.“ (W6M-WL17)
   Translation: "Hello, I have received your flyer about allergies and would like to take part. My daughter is 5 weeks old and I have allergies, so I am very interested.
3. „bei unserer Kinderärztin lag eine Anzeige aus, dass Sie Studienteilnehmer mit Babys und Kleinkindern suchen. Ist dies noch aktuell?“ (W4M-LJ01)
   Translation: "there was an advertisement at our paediatrician's that you were looking for study participants with babies and toddlers. Is this still current?"
4. „Unsere Tagesmutter hat uns auf diese Studie aufmerksam gemacht und wir würden uns hiermit gerne als Teilnehmer für die Studie anmelden“ (W6M-DI06)
   Translation: "Our Nanny made us aware of this study and we would hereby like to register as participants in the study".

1. „Ich habe über meinen Kindergarten Ihr Studienanliegen weitergeleitet bekommen. Meine Tochter ist am xx.xx.xxxx geboren und hat keine Allergien. Gerne nehmen wir an der Studie teil.“ (W6M-DI-14)
   Translation: “I have been forwarded your study request via my kindergarten. My daughter was born on xx.xx.xxxx and has no allergies. We would be happy to take part in the study."

1. „Wir haben in unserer Kinderarztpraxis von ihrer Studie zu Allergien erfahren und würden gerne teilnehmen.“ (W6M-DC79)
   Translation: "We heard about their allergy study in our paediatric practice and would like to participate."
2. „Über den DAAB habe ich von der Studie und der Suche nach einem Elternbeirat gehört. Beides ist für mich sehr interessant, da ich selbst zwei Kinder mit Allergie habe, eines knapp zwei Jahre.“ (SCC-E10)
   Translation: “I heard about the study and the search for a parents' council through the DAAB. Both are very interesting for me, as I have two children with allergies myself, one just under two years old."
3. „Über Twitter habe ich über die Studie erfahren und würde gerne mitmachen und einen Fragebogen zu dem Thema ausfüllen. Ich habe einen 3 Monate alten Sohn.“ (W6M-HS28)
   Translation: "I found out about the study via Twitter and would like to take part and complete a questionnaire on the subject. I have a 3-month-old son."

1. „Ihre Anzeige bei Facebook habe ich gelesen und bin daran sehr interessiert. Zudem erfülle ich alle Kriterien. Sollten Sie noch Teilnehmer suchen, wurde ich mich sehr auf eine Rückmeldung freuen!“ (W6M-SE09)
   Translation: "I read your ad on Facebook and am very interested. I also meet all the criteria. If you are still looking for participants, I would be very happy to hear from you!"

1. „Ich habe soeben Ihren Aufruf im Radio gehört. Das Thema finde ich super. Wir würden sehr gerne teilnehmen.“ (W6M-SE16)
   Translation: "I have just heard your appeal on the radio. I think the topic is great. We would love to participate."

1. „Hallo, ich habe heute ihren Spot bei Radio Regenbogen wegen der Umfrage zu Allergien gehört. Wir sind eine vierköpfige klassische Familie mit Mama, Papa und zwei Mädels und möchten gerne an ihrer Umfrage teilnehmen.“ (W6M-WL15)
   Translation: "Hello, I heard your spot on Radio Regenbogen today about the allergy survey. We are a classic family of four with mum, dad and two girls and would like to take part in their survey."
2. „Ich wurde von meiner Hebamme auf Ihre Studie zur Allergieprävention aufmerksam und würde mich gerne als Teilnehmerin anbieten. Ich habe zwei Kinder. Meine Tochter ist schon 5 Jahre alt, aber mein Sohn ist gerade 3 Monate.“ (W4M-LJ07)
   Translation: "I was made aware of your allergy prevention study by my midwife and would like to offer myself as a participant. I have two children. My daughter is already 5 years old, but my son is just 3 months."
3. -
4. „Ich muss aber ehrlich sagen, ich wüsste nicht, wie mein Partner auf diese Studie hätte aufmerksam werden sollen, weil ich selber bin darüber nur durch ein Forum aufmerksam geworden und habe mich dadurch beteiligt.“ (W4I-FG9)

Translation: "But I have to be honest, I don't know how my partner would have become aware of this study, because I myself only became aware through a forum and got involved through that."

1. „Und diese Dame hat eben eine Facebookgruppe nur für ihre quasi Kursteilnehmerinnen
   I: Ja.
   B: und dort können Fragen gestellt werden, die Hebamme beantwortet die (unv.). Das, finde ich, ist eine super Quelle.
   I: Sie meinen damit auch Notdiensthebamme, oder?
   B: Ja.
   I: Ja, darüber sind Sie auch auf unsere Studie gekommen.
   B: Ach so, ja stimmt, ja, genau. Das ist lustig. Ja, die Kooperation darüber, genau. Die fand ich super.“ (W4I-E11)

Translation: "And this lady just has a Facebook group just for her quasi students.

I: Yes.

B: and questions can be asked there, the midwife answers. I think that's a great source.

I: You mean emergency midwife as well, right?

B: Yes.

I: Yes, that's also how you came to our study.

B: Oh, that's right, yeah, exactly. That's funny. Yes, the cooperation about it, exactly. I thought that was great."

1. „Ich bin über Facebook auf Ihre Umfrage zum Thema „Gesund aufwachsen Kinder gegen Allergien stark machen“ gestoßen. Falls ihr noch Probanden sucht, würde ich gerne daran teilnehmen. Bei Fragen zögern Sie nicht mich per Email oder telefonisch zu kontaktieren.“ (W6M-EV06)

Translation: "I came across your survey on "Growing Up Healthy Making Kids Strong Against Allergies" through Facebook. If you are still looking for subjects, I would love to participate. If you have any questions, please don't hesitate to contact me by email or phone."

**2. Appraisal of credibility, utility**

1. „Uns ist es nur wichtig, dass unsere privaten Daten nicht unverschlüsselt mit Mitarbeitern etc. geteilt werden, z.B. in einer Exceldatei per Email verschickt.“ (W4M-LJ02)
   Translation: "It's only important to us that our private data is not shared unencrypted with co-workers etc., e.g. sent in an excel file via email."
2. „Werden persönliche Daten wie Namen und Geburtsdaten erfragt?“ (W4M-LJ03)
   Translation: "Are personal data such as names and dates of birth requested?"
3. „Ich melde mich wegen den Studien bezüglich Allergien. Interesse habe ich schon. Mein Kind ist hochwahrscheinlich davon betroffen. Können Sie mir mehr dazu sagen: Welche Daten Sie von uns benötigen, was wird eigentlich getestet, Ablauf usw?“ (W4M-LJ04)
   Translation: "I'll get back to you about the allergy studies. I am already interested. My child is very likely to be affected. Can you tell me more about it: what data you need from us, what is actually being tested, procedure, etc.?"
4. "Woher kommt das Geld und wird es wirklich ausgezahlt?"
   Translation: “Where does the money come from and is it really paid out?” (W6T-DC02)
5. "Gibt es einen Haken, verpflichte ich mich jetzt zu etwas?"
   Translation: “Is there a catch, am I now committing myself to something?” (W6T-SA08)

1. „Ich habe im Radio von Ihrem Projekt gehört. Die Studie ist anonym? Dann würde ich gerne mitmachen.“ (W6M-SE24)
   Translation: "I heard about your project on the radio. Is the study anonymous? Then I would like to take part."
2. „Es hilft mir sehr, wenn jemand anderes, dem ich vertraue, die Teilnahme empfiehlt. Dann muss ich keine Zeit damit verschwenden, mich über die Vertrauenswürdigkeit zu informieren"
   Translation: “It helps me a lot when someone else I trust recommends participation. Then I don’t need to waste time on finding out about its trustworthiness” (W3T-P09)
3. „über den [...] erfuhr ich davon, dass Sie Teilnehmer:innen für eine Studie zum Thema Allergien bei Kindern suchen. Da wir für unseren Sohn (9 Monate) kürzlich mehrere positive Allergietests erhielten, könnte ich mir vorstellen, dass eine Teilnahme für uns vielleicht sinnvoll sei (je nach Termin). Ich würde mich daher sehr freuen, wenn Sie uns mehr Informationen zusenden könnten! , z.B. darüber wie die videogestützte Begleitung aussehen würde - *muss* man dies in Anspruch nehmen? Mit einem Kleinkind könnte ich mir halt vorstellen, dass wir nicht 3x 45 Minuten am Stück am Rechner sitzen können, sondern den Fragebogen eher gerne ab und an mal "zwischendurch" (und damit vermutlich "alleine") ausfüllen würden.“ (W4M-LJ24)
   Translation: "I learned through [...] that you are looking for participants for a study on the subject of allergies in children. Since we recently received several positive allergy tests for our son (9 months), I could imagine that participation might make sense for us (depending on the date). I would therefore be very pleased if you could send us more information, e.g. what would video-assisted support look like - do we have to make use of it? With a toddler, I could imagine that we would not be able to sit at the computer for 3 x 45 minutes at a time, but would rather like to fill out the questionnaire "in between" (and thus presumably "alone") from time to time.!"
4. „anbei mein ausgefülltes Formular. Ich hoffe ich konnte mit meinen Antworten sinnvoll zu der Studie beitragen.“ (W4M-LJ29)
   Translation: "Enclosed is my completed form. I hope I was able to contribute meaningfully to the study with my answers."
5. „Ich finde es wundervoll, dass Sie Ursachenforschung betreiben und den Kindern wirklich helfen wollen.“ (W6M-HS28) "I think it's wonderful that you are doing research about the cause and to really want to help the children."
6. „Ich mache mit, weil ich wissen will, stimmt das so was ich denke?“ (W4I-FG12)
   Translation: "I'm taking part because I want to know, is what I'm thinking true?"
7. „Ich habe den Aushang zu der Studie bei unserem Kinderarzt entdeckt. Unsere Tochter ist 19 Monate alt und hat mehrere Lebensmittelallergien (Ei, Nüsse und Milch). Wir haben bereits eine stationäre Lebensmittelprovokation gemacht. Gerne teile ich unsere Erfahrungen um die Forschung zu Allergien bei Kindern zu unterstützen.“ (W4M-LJ28)
   Translation: "I found the notice about the study at our paediatrician's office. Our daughter is 19 months old and has several food allergies (egg, nuts and milk). We have already done an inpatient food provocation. I am happy to share our experience to support research on allergies in children."
8. „Sehr gerne würde ich Sie mit meiner Erfahrung und meinem Wissen unterstützen. Ich bin Mutter von drei Kindern im Alter von 7, 5 und 2 Jahren. Meine mittlerer Sohn leidet unter einer Nussallergie mit Anaphylaxie, Asthma Bronchiale sowie Neurodemitis und einer Hausstaubmilbenallergie. Mein jüngster Sohn hat ein hypersensibles Bronchialsystem.“ (SCC-E08 )
   Translation: "I would be very happy to support you with my experience and knowledge. I am a mother of three children aged 7, 5 and 2. My middle son suffers from nut allergy with anaphylaxis, bronchial asthma as well as neurodemitis and a dust mite allergy. My youngest son has a hypersensitive bronchial system."
9. „Das Abrechnungsformular werden wir nicht nutzen. Aus eigener Erfahrung wissen wir wie zäh und zermürbend das Leben mit Neurodermitis und Allergien ist. Wir hoffen mit unseren Antworten zu helfen und für die Zukunft Familien Linderung zu schenken.“(W6M-398)
   Translation: "We will not use the billing form. We know from our own experience how tough and gruelling life with neurodermatitis and allergies is. We hope to help with our answers and give relief to families for the future."
10. „Durch die vielen negativen Erfahrungen im Umgang mit allergischen Kindern bin ich aber sehr an einer Verbesserung des Systems interessiert und beteilige mich bei Bedarf und zeitlicher Kapazität gerne an Ihrem Projekt.“ (SCC-E07)
    Translation: "Due to the many negative experiences in dealing with allergic children, however, I am very interested in improving the system and would be happy to participate in your project if required and if I have the time capacity."

**3. Interest
 a) thematic content**

1. „Ich möchte hiermit mein Interesse an der Teilnahme der Umfrage zu "Welche Rolle spielt das Thema Allergien im Alltag für Eltern und werdende Eltern?" bekunden. Wir haben eine 2-jährige Tochter mit multiplen Allergien. Deshalb spielt das Thema in unserem Alltag eine große Rolle. Bitte senden Sie mir Informationen bzw. den Link zur Umfrage. Mein Mann wird wahrscheinlich auch teilnehmen.“(W6M-DI11)
   Translation: "I would like to express my interest in participating in the survey on "What role does the topic of allergies play in everyday life for parents and parents-to-be?". We have a 2-year-old daughter with multiple allergies. Therefore, the topic plays a big role in our everyday life. Please send me information or the link to the survey. My husband will probably also take part."
2. „Gerne melde ich mich für die Heuschnupfenstudie an“ (W6M-ID23, W6M-GL21)

Translation: "I would like to register for the hay fever study".

1. „Anmeldung zur Asthma-Studie“ (W6M-SA03)

Translation: "Registration for the Asthma Study"

1. „hiermit möchte ich mein Interesse an der Studie zu Allergien bekunden.“ (W4M-LJ17)
   Translation: "I would hereby like to express my interest in the study on allergies.”
2. „Ich bin selbst betroffen, habe einen sechs Jahre alten Sohn bei dem wir auch eine Allergie vermuten und vor zwei Monaten ist unsere Tochter geboren. Das Thema beschäftigt uns gerade deshalb sehr..“ (W4M-LJ18) "I am affected myself, have a six-year-old son who we also suspect has an allergy and our daughter was born two months ago. That's why we`re dealing a lot with this topic right now...".
3. „Ich bin [...]. Ich habe auf meine digitale Hebammes Webseite über Ihre Studie gelesen. Mein Sohn ist 17 Tage alt :) Ich würde gerne an Ihre Studie teilnehmen. Da mein Mann Laktose Unverträglichkeit hat, das Thema interessiert mich sehr. Auch mein Manns Bruder hat Gluten und Laktose Unverträglichkeit, es war so schlimm, als er 16 war hat der Arzt gesagt er wird nicht mehr groß werden wegen diese Allergien.“ (W4M-LJ19)
   Translation: “I am [...]. I read about your study on my digital midwife website. My son is 17 days old :) I would like to participate in your study. As my husband is lactose intolerant, I am very interested in this topic. Also my husbands brother has gluten and lactose intolerance, it was so bad when he was 16 the doctor said he will not grow up anymore because of these allergies.
4. „meine Hebamme informierte mich über Ihr Forschungsprojekt und ich möchte gerne an Ihrer Studie teilnehmen. Ich würde mich freuen, wenn Sie mir kurz beschreiben, wie der genaue Ablauf sein wird. Auf die Aufwandsentschädigung von 50 Euro würde ich gerne verzichten, wenn das möglich ist. Ich selbst leide schon mein Leben lang an Neurodermitis und ich freue mich über jede Studie, die anderen Menschen (und mir selbst eventuell) helfen kann.“ (W4M-LJ20)
   Translation: "my midwife informed me about your research project and I would like to participate in your study. I would be pleased if you could briefly describe to me what the exact procedure will be. I would gladly waive the expense allowance of 50 euros if that is possible. Me, myself have suffered from neurodermatitis all my life and I am happy about any study that can help other people (and possibly myself)."
5. „Hallo, Ich würde gerne nähere Informationen zu Ihrer Studie erfragen. Da mein Mann und ich beide Allergien und ein 7 Wochen altes Baby haben, ist das Thema Allergieprävention bei uns gerade sehr aktuell.“ (W4M-LJ21)
   Translation: "Hello, I would like to ask for more information about your study. As my husband and I both have allergies and a 7-week-old baby, the topic of allergy prevention is an important one for us at the moment."
6. „über die Hebamme [...] bin ich auf Sie und Ihre Studie aufmerksam geworden. Mein Entbindungstermin ist zwar erst am [...], aber eventuell kann ich ja nach der Geburt noch teilnehmen? Ich finde die Studie sehr interessant, da ich selbst von diversen Allergien geplagt bin und leider keine Behandlungsmethode bislang effektiv dagegen anschlug. Gerne würde ich meinem Kind helfen, dass es nicht später an Allergien leiden muss.“ (W4M-LJ22)
   Translation: "I became aware of you and your study through the midwife [...]. My due date is not until [...], but maybe I can still participate after the birth? I find the study very interesting because I am affected by various allergies and unfortunately no treatment method has been effective so far. I would like to help my child so that it does not have to suffer from allergies later on.
7. „Da ich in [...] wohne kann ich an dem Treffen leider nicht teilnehmen, aber hätte ggf. Interesse, anderweitig zu unterstützen. Kurz zu mir: Ich bin promovierte Molekularbiologin und habe einen einjährigen Sohn mit Neurodermitis bzw. diversen Nahrungsmittelallergien. Daher interessiert mich die Thematik quasi sowohl aus privater als auch aus beruflicher Sicht (auch wenn ich gar nicht mehr als Biologin tätig bin, sondern als Produktmanagerin...).“ (SCC-E05)
   Translation: "As I live in Cologne, I am unfortunately unable to attend the meeting, but I might be interested in supporting in other ways. Briefly about me: I have a doctorate in molecular biology and have a one-year-old son with neurodermatitis and various food allergies. Therefore, I am interested in the topic from a private as well as a professional point of view (even though I am no longer working as a biologist, but as a product manager...)".
8. „Ich bin Mutter eines Babys (3-4 Monate) mit Verdacht auf Kuhmilcheiweissallergie und Mutter einer 4Jährigen mit multiplen Allergien und allergischem Asthma (Anaphylaxierisiko auf Nüsse, Kuhmilcheiweiss, Hausstaub). Die Allergieprävention spielt für mich eine zentrale Rolle (und ist für mich eine Herzensangelegenheit). Sehr gerne möchte ich mich an Ihrem Projekt beteiligen.“ (SCC-E03 )
   Translation: "I am a mother of a baby (3-4 months) with suspected cow's milk protein allergy and a mother of a 4-year-old with multiple allergies and allergic asthma (risk of anaphylaxis to nuts, cow's milk protein, house dust). Allergy prevention plays a central role for me (and is a matter close to my heart). I would very much like to participate in your project."
9. „Über den DAAB habe ich von der Studie und der Suche nach einem Elternbeirat gehört. Beides ist für mich sehr interessant, da ich selbst zwei Kinder mit Allergie habe, eines knapp zwei Jahre. Leider könnte ich in Regensburg nicht dabei sein, freue mich aber, wenn ich dennoch berücksichtigt werden kann oder anderweitig zur Studie beitragen kann.“ (SCC-E10)
   Translation: "I heard about the study and the search for a parents' council through the DAAB. Both are very interesting for me, as I myself have two children with allergies, one just under two years old. Unfortunately, I couldn't be there in Regensburg, but I'm happy if I can still be considered or otherwise contribute to the study."
10. „Ich habe Interesse an der Mitarbeit im Elternbeirat Frühkindliche Allergieprävention. Mein Sohn ist am [...] geboren & hat diverse Lebensmittelallergien, Asthma & eine Eosinophile Ösophagitis. Entsprechend habe ich mich in den letzten drei Jahre sehr viel mit dieser Thematik beschäftigt.“ (SCC-E03)
    Translation: "I am interested in joining the Early Childhood Allergy Prevention Parent Council. My son was born on [...] & has various food allergies, asthma & eosinophilic oesophagitis. Accordingly, I have been very involved with this issue for the past three years."
11. „Wir haben 3 Kinder (6, 4 und 1 Jahr alt) unser 4jähriger Sohn hat eine Erdnussallergie. Deswegen interessieren wir uns sehr für die Allergieprävention und würden gerne an ihrem Elternbeiratstreffen teilnehmen.“ (SCC-E01)
    Translation: "We have 3 children (6, 4 and 1 year old) our 4-year-old son has a peanut allergy. Because of this we are very interested in allergy prevention and would love to attend their parent council meeting."
12. „Ich bin Diaebetologin in [...] und habe 2 Töchter im Alter von 2,5 Jahren und 8 Monaten. Ich interessiere mich persönlich wie auch fachlich sehr für dieses Thema (werde im Jänner auch die Kompaktausbildung zur allergologisch geschulten Ernährungsfachkraft des daab absolvieren). Wenn ich auf Grund meiner Profession nicht zur Zielgruppe Ihrer Forschung gehöre, teilen Sie es mir bitte mit. Ansonsten würde ich mich freuen, wenn ich Ihnen als Mutter weiterhelfen kann.“ (SCC-E02 )
    Translation: "I am a dietician in [...] and have two daughters aged 2.5 years and 8 months. I am very interested in this topic, both personally and professionally (in January I will also complete the daab's compact training course for allergy-trained nutritionists). If I do not belong to the target group of your research due to my profession, please let me know. Otherwise, I would be happy to help you as a mother."
13. „Ich würde liebend gerne an dieser Studie teilnehmen. Ich bin deshalb so interessiert, da ich selbst an Allergien leide.“ (W6M-EV08)
    Translation: "I would love to take part in this study. I am so interested because I suffer from allergies myself."
14. „Ich interessiere mich im Allgemeinen sehr für das Thema Allergien von Kindern habe mich aber noch nicht so sehr damit befasst. Ich habe einige Allergien wie z.B. Hausstaub, verschiedene Lebensmittel, Beifuß und Lebensmittelintolleranzen. Meine Kinder sind 5 und 3 und immer mal wieder mache ich mir einfach Sorgen....gerade, wenn die Wespen und Bienen wieder viel unterwegs sind oder auch damals als meine Kinder zum ersten Mal Erdbeeren gegessen haben. Immer wenn etwas Neues kommt, bin ich etwas ängstlich und weiß nicht wie ich da am besten ran gehen soll. Bisher haben sie noch keine Allergie entwickelt, aber es könnte ja doch jederzeit so weit sein.“ (W6M-HR11)
    Translation: “I am generally very interested in the topic of children's allergies but have not yet dealt with it that much. I have a few allergies such as house dust, various foods, mugwort and food intolerances. My children are 5 and 3 and every now and then I just worry....just when the wasps and bees are around a lot again or also when my children ate strawberries for the first time. Whenever something new comes along, I'm a bit anxious and don't know how best to approach it. So far they haven't developed an allergy, but it could happen at any time.

1. „Falls Sie noch Teilnehmer benötigen, würde ich mich bereit erklären. Ich bin Mutter von 2 Söhnen. Der eine wird Anfang Juli 1 Jahr alt und der andere Ende August 3 Jahre. Mein Mann hat eine Hausstauballergie sowie eine Allergie gegen Gräser, Pollen etc. Bei mir selbst ist keine Allergie bekannt, aber ich finde das Thema sehr interessant, da ich ab und an schon das Gefühl hatte, dass bei meinem Großen im Bett die Nase zu geht und ihn gerne mal auf Allergien testen lassen würde.“ (W6M-HS17)
   Translation: "If you still need participants, I would be willing to participate. I am a mother of 2 sons. One will be 1 year old at the beginning of July and the other 3 years old at the end of August. My husband has a house dust allergy as well as an allergy to grasses, pollen, etc. I don't have any known allergies myself, but I find the topic very interesting, as I have had the feeling from time to time that my big boy's nose closes up in bed and would like to have him tested for allergies."
2. „Mein Sohn ist momentan 4 Monate alt. Mama und Papa haben Allergien sowie Nahrungsmittelunverträglichlkeit. Aus Interesse für mich und den Rest der Familie und um bei dieser Studie mitzuwirken melde ich mich nun bei ihnen.“ (W6M-WL18)
   Translation: "My son is currently 4 months old. Mum and dad have allergies as well as food intolerances. Out of interest for me and the rest of the family and to participate in this study, I am now contacting them."
3. „Unser Sohn ist 7,5 Monate alt und bisher sind bei ihm keine Allergien bekannt. Allerdings gibt es auf meiner Familienseite einige bekannte und auch starke Allergiker. Daher habe ich ein besonderes persönliches Interesse am Erfolg Ihrer Studie.“ (W6M-WL21)
   Translation: "Our son is 7.5 months old and so far he has no known allergies. However, there are some known allergy sufferers on my side of the family and also severe allergy sufferers. Therefore, I have a special personal interest in the success of your study."
4. „Sollten Sie noch Teinehmer suchen, würde ich gerne mitmachen. Meine Frau auch. Beide sind wir Allergiker. Unsere Tochter ist aktuell 22 Monate alt und wir vermuten auch bei ihr schon Anzeichen für Allergien.“ (W6M-EV17)

Translation: "If you are still looking for participants, I would love to join. My wife too. We are both allergy sufferers. Our daughter is currently 22 months old and we suspect she is already showing signs of allergies as well."

1. „Wir haben zwei Kinder, Sohn 19 Monate, Tochter 4 Monate. Ersterer ist gerade von Nesselsucht betroffen, Blutergebnisse brachten nichts. Studienanfrage kommt passend.“ (W6M-GL10)

Translation: "We have two children, son 19 months, daughter 4 months. The former is just affected by hives, blood results yielded nothing. Study request comes appropriate."

1. „sehr gerne möchte ich an Ihrer Studie im Rahmen eines telefonischen Einzelgespräches teilnehmen. Unsere Tochter ist seit heute 7 Wochen alt und hat aufgrund der Dispositionen seitens meines Mannes und mir (diverse Allergien, Neurodermitis, Asthma) ein hohes Potential hierfür.“ (W4M-LJ23)

Translation: "I would very much like to participate in your study in a one-on-one telephone interview. Our daughter is 7 weeks old as of today and has a high potential for this due to dispositions on the part of my husband and me (various allergies, neurodermatitis, asthma)."

1. „Ich habe Interesse an der Studie teilzunehmen. Meine Kinder sind 14 Monate und fast 3 Jahre alt. Bitte lassen Sie mir weitere Informationen zukommen.“ (W4M-LJ25)

Translation: "I am interested in participating in the study. My children are 14 months and almost 3 years old. Please let me know more information."

**b) Supportive:**

1. „Ich habe selber meine Bachelor-Arbeit an der Hochschule durchgeführt. Ich freue mich, wenn ich mit meiner Teilnahme etwas zurückgeben kann.“ (W6T-SA10)
   Translation: "I did my bachelor’s thesis at the university myself. I am happy if I can give something back with my participation."
2. „Wir haben von unserer Tagesmutter die Informationen zu Ihrer Studie bekommen. Da ich selbst an der PH Freiburg studiert habe, würde ich gerne zusammen mit meinem Mann an der Studie teilnehmen, um sie zu unterstützen.“ (W6M-DI13)

Translation: “We received the information from our childminder. Since I studied at PH Freiburg myself, I would like to take part in the study together with my husband to support her.”

1. „Ich könnte mir vorstellen an Ihrer Studie teilzunehmen. Meine Tochter wird im August 2, hat vermutlich eine Allergie gegen Spritzmittel ist aber ansonsten Allergie frei. Ihr 5-jähriger Bruder hat keine Allergien und ich habe Allergien gegen Tierhaare und Heuschnupfen. Ich denke das könnte gut passen, bzw. Wir ganz hilfreich sein. [...] habe ich selber meinen Abschluss an der PH gemacht, da helfe ich doch gerne der Heimat.“ (W6M-SE08)

Translation: “I could imagine taking part in your study. My daughter will be 2 in August, she probably has an allergy to sprays but is otherwise allergy free. Her 5-year-old brother has no allergies and I have allergies to animal hair and hay fever. I think this could be a good fit, or we could be quite helpful. I graduated from PH myself in 2011, so I'm happy to help out.”

1. „Gerne nehme ich an der Studie teil. Wenn wir aus der Pandemie etwas lernen, dann doch wie wichtig die Wissenschaft ist.“ (W6M-GL07)

Translation: "I would be happy to participate in the study. If we learned anything from the pandemic, it's how important science is."

1. „Ja super. Also klingt total spannend, klingt total interessant und ich freue mich auch, wenn man selber vielleicht so einen kleinen, kleinen, kleinen Beitrag dazu leisten kann und komplett nochmal was (unv.) kann.“ (W4I-E14)

Translation: „Great. So it sounds totally exciting, totally interesting, and I'm also happy if you can perhaps make a small, small, small contribution to it yourself and completely do something again.“

1. „Wir möchten uns gerne zur Teilnahme an der Allergie Studie anmelden und freuen uns, unseren Beitrag leisten zu können.“ (W6M-SE17)

Translation: „We would like to register to participate in the allergy study and look forward to making our contribution.“

1. „Meine ältere Tochter hat Allergien und mir ist es sehr wichtig Sie bei dieser Studie zu unterstützen (auch ohne Entgeld und wenn nur die Daten zum jüngeren Kind verwendet werden können, da das ältere bereits 6 Jahre alt ist). Ich finde, dass man sehr wenig Informationen zu Prävention/Umgang mit Allergien bei Kindern findet.“
   Translation: "My older daughter suffers from allergies, and it is very important to me to support you in this study (even without payment and if only the data on the younger child can be used, since the older one is already 6 years old). In my opinion, little information is available on the prevention and management of allergies in children." (W6M-WL20)
2. „Ich habe eine 14-monatige Tochter habe und würde gerne an eurer Studie teilnehmen und euch unterstützen.“ (W6M-HA1)

Translation: "I have a 14-month-old daughter and would like to participate in your study and support you."

1. „nochmal vielen Dank für das schöne Gespräch gestern Abend! Ich hoffe, ich konnte hilfreich sein für Ihre Studie.“ (W4M-LJ26)

Translation: "thank you again for the lovely conversation last night! I hope I was able to be helpful in your study."

1. „Unser Sohn [...] ist 7,5 Monate alt und bisher sind bei ihm keine Allergien bekannt. Allerdings gibt es auf meiner Familienseite einige bekannte und auch starke Allergiker. Daher habe ich ein besonderes persönliches Interesse am Erfolg Ihrer Studie.“
   Translation: Our son [...] is 7.5 months old and so far he has no known allergies. In my family, there are strong allergy sufferers. That’s why I’m keen in the success of your study” (W6M-EV21)
2. „Wir betreuen unsere Kinder derzeit zuhause. Keines der Kinder hat Allergien. Als Tagesmutter bin ich aktuell aufgrund der Schwangerschaft noch nicht aktiv. Das werde ich aber nächstes Jahr aufgreifen. Mich interessiert daher sowohl aus privaten als auch aus beruflichen Gründen die Studie sehr. Vor allem bin ich an den Hypothesen und Erkenntnissen im Rahmen der Studie interessiert. Sehr gerne leiste ich/leisten wir daher unseren Beitrag zu Ihrer Studie und freuen uns über Ihre Rückmeldung.“ (W6M-GL15)

Translation: "We currently lookg after our children at home. None of the children have allergies. I am not currently active as a nanny because of my pregnancy. But I will take that up next year. I am therefore very interested for both private and professional reasons. Above all, I am interested in the hypotheses and findings of the study. I am/we are therefore very happy to make our contribution and look forward to your feedback."

1. „Ich habe heute einen Beitrag der Hochschule Freiburg im Radio gehört und würde gerne mehr Informationen zur Umfrage bezüglich Allergien bei Kindern bekommen, um Sie ggf. bei Ihrer Arbeit und dem Weg zu weniger Allergien unterstützen.“ (W6M-WL16)

Translation: "I heard a piece from Freiburg University of Applied Sciences on the radio today and would like to get more information about the survey regarding allergies in children to help you with your work and the path to fewer allergies, if necessary."

**c) gratification:**

1. „Ich bin zeitlich ziemlich flexibel, da ich meine Hausbesuche auch kurzfristig durchführen kann. Ich habe mich jedoch morgen von 8:30 Uhr bis 15 Uhr zu einer FoBi (Online) angemeldet. Die 50 Euro Aufwandsentschädigung können Sie gerne in Ihre „Kaffeekasse“ stecken. Wie findet das Interview statt? Wenn Ihnen Skype oder Zoom eher mehr helfen würde, wäre das auch kein Problem. Benötigen Sie im Vorfeld noch irgendwelche Angaben von mir?“ (W3M-M08)
   Translation: "I am quite flexible in terms of time as I can do my home visits at short notice. However, I have signed up for a FoBi (online) tomorrow from 8:30 am to 3 pm. You are welcome to put the 50 euros expense allowance into your "coffee fund". How will the interview take place? If Skype or Zoom would help you more, that would be no problem. Do you need any more information from me in advance?"
2. „ich würde mich bereit erklären, ein Interview zu führen welches zu 30 Euro vergütet wird. Ich könnte immer zwischen 13 und 14 Uhr, wenn mein Kleiner sicher schläft telefonieren. Sie können mir gern 1-2 Terminvorschläge schicken.“ (W4M-LJ31)
   Translation: "I would be willing to do an interview which would be paid at 30 euros. I could always call between 1 and 2pm when my little one is safely asleep. You are welcome to send me 1-2 appointment suggestions."
3. „ich habe Ihr Formular bezüglich der Aufwandsentschädigung erhalten und habe freudig zur Kenntnis genommen, dass Sie handschriftlich die 30€ in 50€ umgewandelt haben. Dafür möchte ich mich besonders bedanken. Ich hatte direkt nach der Teilnahme an der Studie im Freundeskreis Werbung gemacht und soweit ich weiß, hat sich meine Freundin xx bei Ihnen gemeldet… sie hatte es zumindest vor teilzunehmen.“ (W4M-LJ63)
   Translation: "I have received your form regarding the allowance and was pleased to note that you have handwritten the 30€ into 50€. I would like to thank you especially for this. I had advertised the study among my friends immediately after participating and as far as I know, my friend xx contacted you... at least she intended to participate."
4. „Nun bin ich fertig mit der Studie und freue mich über die 30€. Gerne dürfen Sie mich wieder Kontaktieren für eine weiter Umfrage.“ (W6M-HA288)
   Translation: "Now I am done with the survey and am happy about the 30€. You are welcome to contact me again for another survey."
5. „Unerwartet habe ich dabei viel über das Thema Allergien und Unverträglichkeiten gelernt. Somit werde ich mehr aus dieser Befragung mitnehmen können als die Aufwandsentschädigung. Vielen Dank!“ (W6M-DC212)
   Translation: "Unexpectedly, I learned a lot about the topic of allergies and intolerances. So I will be able to take away more from this survey than the allowance. Thank you very much!"

1. „Ja, die Befragungen haben Spaß gemacht und waren zusätzlich auch ein gutes
   Gehirntraining. ;)“ (W6M-SA125)
   Translation: "Yes, the surveys were fun and also good a brain training. ;)"
2. „Vielen Dank für die Teilnahme der Studie. Es war wirklich sehr interessant und auch etwas für die grauen Zellen dabei, was mir sehr gefallen hat.“ (W6M-HA411)
   Translation: "Thank you very much for participating in the study. It was really very interesting and also something for the grey cells, which I really enjoyed."
3. „Vielen Dank für die tolle Umfrage ich habe in vielen Bereichen dann das Nachdenken angefangen wo ich mir sonst nicht so die Gedanken Gemacht habe.“ (W6M-SA131)
   Translation: "Thank you very much for the great survey, I have started thinking in many areas where I would not otherwise have thought about it."

1. „ich schreibe jetzt zum 3. Mal, hatte Ihnen meine Teilnahmebescheinigung am Interview mit Frau [...] geschickt und warte immer noch auf die Aufwanfsentschädigung von 50 Euro. Bitte erfreuen Sie mich doch noch damit, Sonst verliere ich das Vertrauen ins UKR und allgemein da rein, wenn mir jemand eine Aufwandsentschädigung für meine Zeit für Umfragen, Interviews und dergleichen anbietet. Anbei noch einmal die Teilnahmebestätigung.“ (W3M-M19)

Translation: "I am now writing for the 3rd time, had sent you my certificate of participation in the interview with [...] and am still waiting for the expense allowance of 50 euros. Please please me yet with it, Otherwise I lose the confidence in the UKR and generally there in, if someone offers me an expense allowance for my time for surveys, interviews and the like. Enclosed once again is the confirmation of participation."

1. „Wir haben Ihre Kontaktdaten über XXX erhalten. Uns wurde gesagt, wir bekommen von Ihnen einen Link zur Teilnahme an einer Umfrage und dafür 30Euro.“ (W6M-HA95)

Translation: "We received your contact information through XXX. We were told we would receive a link from you to participate in a survey in exchange for 30Euro."

**4. Participation of people in the social environment**

1. „eine Mutti [...] hat berichtet, dass sie noch Familie für ihre Studien als Interviewteilneher suchen. Wir würden uns hier gerne beteiligen. Wir haben zwei Kinder im Alter von 5 und 4 Monaten.“ (W4M-LJ05)
   Translation: "A mum [...] has reported that they are still looking for familys for their studies as interview participants. We would love to participate here. We have two children aged 5 and 4 months."
2. „ich bin eine Freundin von [...] und [...] (ebenfalls mit kleinem Kind) und [...] hatte mir den Flyer zur Studie weitergeleitet und gesagt, dass ihr noch Familien sucht, die mitmachen. Wenn dem immer noch so ist, würde ich mich gerne bereit erklären.“ (W4M-LJ06)
   Translation: "I am a friend of [...] and [...] (also with a small child) and [...] had forwarded me the flyer about the study and said that you were still looking for families to participate. If that is still the case, I would be happy to volunteer."
3. „Ich bin durch meine Freundin [...] (kooperierende Universität) auf eure Studie aufmerksam geworden und würde gerne teilnehmen.“ (W6M-WL02)
   Translation: "I became aware of your study through my friend [...] (cooperating university) and would like to participate."
4. „Ich habe heute von einer Bekannten von der Studie erfahren. Mein Jüngster ist 3 Jahre alt (20.10.2017) sind wir für die Studie von Interesse?“ (W6M-GL02)
   Translation: "I heard about the study today from an acquaintance. My youngest is 3 years old (20.10.2017) are we of interest for the study?"
5. „Ich habe von meiner Schwester von der tollen Umfrage gehört und würde gerne daran teilnehmen mit meinem Partner, da wir uns sehr für Allergien usw. interessieren!“ (W6M-SA76)
   Translation: "I heard about the great survey from my sister and would love to take part with my partner as we are very interested in allergies etc!"
6. „ich bin dran, habe heute mit einem guten Freund, Herrn Dr. med.xxx, telefoniert. Er ist bereit, an der Befragung teilzunehmen und ist unter der Mobilfunknummer xxx für Sie erreichbar.“ (W3M-P03)
   Translation: "it's my turn, I spoke to a good friend, Dr.med.xxx, on the phone today. He is willing to take part in the interview and can be reached at the mobile number xxx for you."
7. „Hallo, ich habe den Kontakt von Dr. [...] bekommen. Ich würde bei dem Interview mitmachen. Wie vereinbaren wir einen Termin?“ (W3M-P20)
   Translation: "Hello, I got the contact from [...]. I'd like to take part at the interview. How do we arrange an appointment?"
8. „Gerne würde ich an Ihrem Interview Teilnehmen. Habe eine Empfehlung von einer Dame aus Instagram erhalten. Ich habe auch 1000 Follower und kann gerne weiter empfehlen, wenn es mir zu sagt.“ (W4M-LJ08)
   Translation: "I would love to take part in your interview. I received a recommendation from a lady on Instagram. I also have 1000 followers and am happy to recommend if it appeals to me."
9. „Ich möchte gerne an der Studie teilnehmen. Bin selbst Tagesmutter und habe es bereits an 10 Eltern weitergeleitet. (W6M-SE06)
   Translation: “I would like to participate in the study. I'm a Nanny myself and have already passed it on to 10 parents.

1. „Ich habe am Montag von unserer Tagesmutter euren Flyer bekommen. Online steht, dass das Teilprojekt 6 im Mai startet. Kann man sich denn jetzt noch dafür anmelden? Dann würden mein Mann und ich das gerne für unseren 22 Monate alten Sohn tun. Und lohnt es sich noch den Aufruf für die Studie an bekannte Eltern weiterzuleiten?“ (W6M-WL09)
   Translation: "I got your flyer from our Nanny on Monday. It says online that sub-project 6 starts in May. Is it still possible to register for it now? If so, my husband and I would like to do it for our 22-month-old son. And is it still worth it, to forward the call for the study to parents we know?"
2. „Und noch eine andere Frage: Soll ich es noch weitersagen, so dass sich evtl. noch jemand bei Ihnen meldet, der auch teilnehmen möchte? Oder haben Sie bereits genügend Teilnehmer?“ (W4M-LJ27)

Translation: "And another question: Should I spread the word so that someone else might contact you who would also like to participate? Or do you already have enough participants?"

1. „ich würde ihr anschreiben und den Flyer an die jeweiligen Teamverantwortlichen der Schulungsteams senden. Ich würde im Text jedoch auch auf die Möglichkeit der direkten Ansprache der Eltern hinweisen. Ob dann Eltern direkt motiviert werden, ist sehr vom einzelnen Team abhängig. Bez. der Fachgesellschaften können sie sich auf mich beziehen, vielleicht ist das hilfreich. Wenn sie mir also den Brief und den Flyer nochmal zusenden, würde ich diesen an die Asthma-Schulungsteams weiterleiten. Wenn rechnen sie mit Ergebnissen? An denen sind wir natürlich interessiert.“ (W4M-LJ16)
   Translation: "I would send your letter and the flyer to the respective team leaders of the training teams. However, in the text I would also refer to the possibility of addressing parents directly. Whether parents are then directly motivated depends very much on the individual team. Regarding the professional associations, they can refer to me, maybe that will be helpful. So if you send me the letter and the flyer again, I would forward it to the asthma training teams. When do you expect to see results? We're interested in those, of course."
2. „Liebe Schulungsteams, von der Medizinischen Hochschule Hannover (MHH) wird in Kooperation mit anderen Hochschulen eine Studie durchgeführt, die herausbekommen möchte, wie sich Eltern von Kindern unter 3 Jahren mit Allergien Informationen beschaffen (möchten). Diese Information ist natürlich auch für uns sehr interessant und bitte sie deshalb diese Studie so gut wie möglich zu unterstützen“ (W4M-LJ30)
   Translation: „Dear training teams, the Hannover Medical School (MHH) is conducting a study in cooperation with other universities to find out how parents of children under 3 with allergies obtain information. This information is of course also very interesting for us and we therefore ask you to support this study as much as possible".
3. „Hallo, wir sind eine 5köpfige Familie. Unsere beiden Kleinen sind (noch) 2 und 5 Jahre alt. Gern würden wir an der Studie zum Thema: Gesundheit und Allergien bei Kindern teilnehmen. Die Information, dass noch Teilnehmer für die Umfrage gesucht werden haben wir von unserem Kindergarten erhalten.“ (W4M-LJ09)

Translation: "Hello, we are a family of 5. Our two little ones are (still) 2 and 5 years old. We would like to participate in the study on the topic: health and allergies in children. We received the information that participants are still wanted for the survey from our daycare."

1. „Ihre vorformulierte Mail mit den Anhängen haben wir heute im [...] -Mitgliederverteiler versendet“ (W4M-LJ10)

Translation: "We sent your pre-written mail with the attachments today in the

[...] member mailing list"

1. „als Vorsitzender der [...] könnte ich den Studienaufruf an die Asthmateams weiterleiten – das sind zum größten teil niedergelassene Pädiater mit einer gewissen Spezialisierung (gut 200 Teams), ich denke die GPA, GPP und BAPP haben sie schon angeschrieben?“ (W4M-LJ11)

Translation: "as chair member of the [...], could I forward the study call to the asthma teams - these are mostly pediatricians in private practice with some specialization (a good 200 teams), I think the GPA, GPP and BAPP have already written to them?"

1. „Mein Mann würde ebenfalls an der Studie teilnehmen. Brauchen Sie hierfür noch weitere Infos?“ (W6M-DC223)

Translation: "My husband would also participate in the study. Do you need any more info for this?"

1. „Ich bin bereit für den 2. Teil. Meine Schwester ist auch schwanger im 7. Monat. Kann/darf Sie da auch mitmachen?“ (W6M-SA44)

Translation: "I am ready for the 2nd part. My sister is also 7 months pregnant. Can she participate in this as well?"

1. „Mir wurde Ihre Studie von XXX weitergeleitet. Ich würde gerne mit meinem Partner und meiner Tochter (6 Monate) daran teilnehmen. Gerne kann ich auch die Informationen in meinem Freundes- und Bekanntenkreis weiterleiten, sofern dies gewünscht ist.“ (W6M- HA063)

Translation: "I was forwarded your study from XXX. I would like to participate with my partner and daughter (6 months). I would also be happy to forward the information to my circle of friends and acquaintances, if desired."

1. „Informationen zur Studie habe ich auch in meinem Freundes-/ Bekanntenkreis verteilt.“ (W6M-HA107)

Translation:"I have also distributed information about the study to my circle of friends/acquaintances."

1. „Ich habe bereits damit angefangen und finde es sehr interessant. Mein Mann würde auch gerne an der Befragung teilnehmen.“ (W6M-HA423)

Translation: "I have already started it and find it very interesting. My husband would also like to participate in the survey."

1. „Ich hab es Freundinnen weitergeleitet, hoffentlich machen sie mit.“ (W6M-HA426)

Translation: "I have forwarded it to friends, hopefully they will participate."

**5. Determining self-efficacy**

1. „durch eine Bekanntin habe ich erfahren, dass Sie Familien mit klein Kind für die Studien suchen. Ich denke ,ich kann da gut passen. Ich bin [...] die Mutter von drei Kindern, die kleinste ist 3 Jahre alt. Ich komme aus [...] und seid 10 Jahren wohne in Deutschland. Ich spreche deutsch aber für mich wäre besser ,wenn das eine Einzelne Gäsprech wird. Wenn Sie Interesse haben ,kann ich gerne an der Studien teilnehmen.“ (W4M-LJ12)
   Translation: "I heard from an acquaintance that you are looking for families with small children for the studies. I think I can fit in well. I am [...], the mother of three children, the youngest is 3 years old. I come from [...] and have been living in Germany for 10 years. I speak German but for me it would be better if this is a one to one conversation. If you are interested, I would be happy to take part in the studies.
2. „Gerne würde ich an der Studie teilnehmen. Ich habe einen 5jährigen Sohn. Ein persönliches Gespräch in Präsenz, alleine oder mit 1 bis 2 Eltern würde ich bevorzugen.“ (W4M-LJ15)
   Translation: "I would like to take part in the study. I have a 5-year-old son. I would prefer a personal interview in presence, alone or with 1 to 2 parents."
3. „Ich bin per Mail erreichbar und würde gerne im Einzelgespräch teilnehmen da ich alleinerziehend bin und hoffe das der kleine zum Zeitpunkt des Interviews schläft...“
   Translation: "I can be reached by email and would like to participate in the one-to-one interview as I am a single parent and hope that the little one will be asleep at the time of the interview...".
4. „Ich nehme gerne an einem Gruppengespräch Teil. Da ich aber ständig meinen kleinen Sohn dabei habe und nicht weiß, ob das störend ist, können wir auch gerne einen telefonischen Termin vereinbaren.“ (W4M-LJ56)
   Translation: "I would be happy to take part in a group discussion. But since I have my little son with me all the time and I don't know if that would be disturbing, we can also make an appointment by phone."
5. „Kann ich trotzdem teilnehmen, obwohl ich nichts über Allergien weiß und niemand in meiner Familie an Allergien leidet?“
   Translation: "May I still participate if I don't know anything about allergies and no one in my family suffers from allergies?" (W6M-HR07)
6. „Ist es denn auch sinnvoll, an der Studie teilzunehmen, wenn unsere Kinder keinerlei Allergien haben?“ (W6M-EV03)
   Translation: "Does it make sense to take part in the study if our children do not have any allergies?"

1. „Leider reicht meine Fantasie nicht aus um mir vorstellen zu können welche Fragen man 3 x 45 Minuten beantworten muss, wenn bei einem Kind keine Allergien vorliegen.“ (W6M-SA14)
   Translation: "Unfortunately, I don't have enough imagination to know what questions you have to answer for 3 x 45 minutes if a child has no allergies."
2. „da ich selbst ausschließlich angestellt arbeite, halte ich mich persönlich nicht für geeignet zum Interview. Leider bin ich auch zeitlich extrem eingeschränkt.“ (W3M-M27)
   Translation: "since I myself work exclusively as an employee, I personally do not consider myself suitable for the interview. Unfortunately, I am also extremely limited in terms of time."
3. „Ich würde mich freuen, wenn Sie mir kurz beschreiben, wie der genaue Ablauf sein wird.“ (W4M-LJ14)

Translation: "I would appreciate a brief description of what the exact process will be."

1. „Also ich finde den Austausch in einer kleinen Gruppe auch gut und von der Uhrzeit ist es auch in Ordnung. Und gerade die Videoanrufe sind wir glaube ich alle jetzt mittlerweile gewöhnt. (lacht) Von dem her ist es auch ein völlig akzeptabler Rahmen.“ (W4I-FG1)

Translation: "Well, I think the exchange in a small group is also good and from the time it is also okay. And especially the video calls I think we are all used to by now. (laughs) From that point of view, it's also a completely acceptable setting."

1. „Und ich dachte, okay, irgendwann will ich es mal angehen, definitiv und mich da halt auch mal mehr informieren. Und wollte das halt, quasi unser Gespräch so ein bisschen als Startschuss nehmen.“ (W4I-E22)

Translation: "And I thought, okay, at some point I want to tackle it, definitely, and just get more information. And wanted to take that just our conversation so a bit as a starting point."

**6. Appraisal of the appropriateness of the effort**

1. „Da ich aus [...] komme, wäre mir eine Teilnahme in Präsenz in [...] für nur 3 Stunden zu zeitaufwändig bezüglich der An & Abreise. Der Kosten-Nutzen-Faktor macht für mich keinen Sinn. Vielleicht wäre eine digitale Teilnahme möglich, wenn Sie eine Hybrid-Veranstaltung daraus machen würden.“ (SCC-E09)
   Translation: "As I come from [...], attending in [...] for only 3 hours would be too time-consuming for me in terms of travelling to and from the event. The cost-benefit factor does not make sense for me. Perhaps digital participation would be possible if you made it a hybrid event."
2. „Ich habe es gerade noch geschaffen, wobei sie Konzentration um diese Zeit natürlich etwas nachgelassen hat. Schade, dass der 3. Teil nicht mehr um Allergien und nur im Corona ging. Ebenfalls Schade ist auch, dass man nicht die Option hat anzugeben, wenn man selber Risikogruppe ist. Das würde nämlich vielleicht erklären, warum ich besonders vorsichtig im Verhalten sein musste... so verfälscht das etwas die Daten, meiner Meinung.
   Weiterhin viel Erfolg für Ihre Studie. Ich habe es nur gemacht, weil ich ebenfalls mal an der ph Freiburg studiert habe. Der zeitliche Aufwand (gerade als mehrfach Mama) ist dafür einfach viel zu hoch und 30€ schon wenig für die kostbare "Freizeit" von denen Mamas sowieso zu wenig Jbd.“ (W6M-HA443)
   Translation: "I just about managed it, though of course her concentration was a bit off at this time. It's a pity that the 3rd part was no longer about allergies and only in Corona. It's also a pity that you don't have the option to indicate if you are a risk group yourself. That would perhaps explain why I had to be particularly careful in my behaviour... In my opinion, this distorts the data somewhat. Good luck with your study. I only did it because I also once studied at the ph Freiburg. The time involved (especially as a multiple mum) is simply far too high and 30€ is not enough for the precious "free time" that mums don't have enough of anyway.”

1. „Ich hätte noch eine Frage: im Flyer zu der Studie wurde noch angekündigt, dass man zum Schluss Infomaterial zum Thema Allergien und deren Vorbeugung im Kindesalter bekommt. Könnten Sie uns es noch zukommen lassen? Das war nämlich die Belohnung, auf die ich mich gefreut hab.“ (W6M-SA161)
   Translation: "I have another question: in the flyer for the study it was announced that at the end you would receive information material on the subject of allergies and their prevention in childhood. Could you send it to us? Because that was the reward I was looking forward to."

**Intention**

**Planning of concrete participation (coping)**

1. „Ich bin auf die Studie im Netz aufmerksam geworden und Interessiere mich für die Teilnahme. Wie genau läuft die Studie ab? Wann wäre das Gespräch und wie kann ich denn Teilnehmen? Ich bin Mutter einer 21 Monate alten Tochter und eines 6 Monate alten Sohnes.“ (W4M-LJ13)
   Translation: "I became aware of the study on the internet and am interested in participating. How exactly does the study work? When would the interview be and how can I take part? I am a mother of a 21-month-old daughter and a 6-month-old son."
2. „ja, der [...] geht. Normalerweise müsste es auch mit der Internetverbindung klappen. Sollte ich doch nicht zu Hause sondern unterwegs sein, ginge es dann auch per Telefon? Falls es per Video wäre, muss ich mich dann einfach um [...] unter dem Link einloggen?“ (W4M-LJ34)
   Translation: "Yes, [...] is possible. Normally it should also work with the internet connection. Should I not be at home but on the road, would it also work by phone? If it's by video, do I just have to log on to the link at [...]?"
3. „ich habe durch eine Bekannte erfahren, dass Sie Interviews anbieten zum Thema Eltern von Kleinkindern und deren Leben in der Pandemie. Ich würde gerne an dem Interview teilnehmen und benötige aber noch einige Informationen. Die wichtigste Frage die ich habe: läuft das Gespräch telefonisch oder persönlich ab?“ (W4M-LJ38)
   Translation: "I learned through an acquaintance that you offer interviews on the topic of parents of young children and their lives in the pandemic. I would like to take part in the interview, but I still need some information. The most important question I have: will the interview take place by phone or in person?"
4. „arbeiten Sie am Mittwoch, den 18.11.20? Da würde es ab 11 Uhr bis abends gut passen. Wie kann ich mich vorbereiten? Um welche Themenbereiche handelt es sich?“ (W3M-M15)
   Translation: "are you working on Wednesday 18.11.20? It would work from 11am until the evening. How can I prepare? What kind of topics are involved?"
5. „Hallo hab diese Karte bekommen das sie Mütter für ihre Studie suchen würde gerne teilnehmen nur hätte ich gerne genauere Informationen wie das läuft kann man es nur online machen den ich habe nur ein Handy und nicht so gutes Internet.“ (W6M-HS13)
   Translation: "Hi, I got this card that they are looking for mothers for your study, I would like to take part but I would like more information on how it works, can you only do it online because I only have a mobile phone and not very good internet."
6. „Wir würden uns für die Studie Kinder gehen Allergien stark machen interessieren? Muss man nur die 3 Fragebogen a 45min ausfüllen oder kommt auf einen mehr zu?“ (W6M-SA01)

Translation: "We would be interested in the study “Kinder gehen Allergien stark machen?” Do you only have to fill out the 3 questionnaires a 45min or is there more to come?"

1. „nun hat sich eine zweite Gruppe von 6 Frauen gesammelt. Wollen Sie sie befragen? Als Termin schlage ich den Di. 28.09.2021 um 12 Uhr vor. Auch beim zweiten Termin werden wir Unterstützung ihrer Kollegin [...] benötigen.“ (W4M-LJ49)
   Translation: "now a second group of 6 women has gathered. Do you want to interview them? I suggest Tues 28/09/2021 at 12 noon as the date. We will also need the support of your colleague [...] for the second appointment."
2. -
3. „10:00 Uhr klingt erstmal passend. Brauchen Sie nur mich für das Interview oder geht es anschließend noch weiter? Ich würde mich über einen kurzen Ablaufplan freuen.“ (W4M-LJ32)

Translation: "10:00 sounds convenient for now. Do you need just me for the interview or does it go on afterwards? I would appreciate a brief schedule."

1. „mein Mann wäre auch sehr gerne dabei. Würde ein anderer Wochentag (Montag, Mittwoch oder Freitag) ebenfalls gehen?“ (W4M-LJ33)

"my husband would also love to be there. Would another day of the week (Monday, Wednesday, or Friday) work as well?"

1. „ich würde an der Studie Gesundheit bei Kleinkindern mitmachen. Meine Tochter ist 15 Monate alt. Wie wäre der Ablauf? Da wir in [...] wohnen müsste es telefonisch oder per Fragebogen erfolgen. Dazu könnte ich am besten morgens um ca. 9 Uhr. Viele Grüße“ (W4M-LJ35)

Translation: "I would participate in the infant health study. My daughter is 15 months old. What would the schedule be like? Since we live in southern Germany it would have to be by phone or questionnaire. I could do this best in the morning at about 9am. Many greetings"

1. „Hallo, von Frau [...] habe ich erfahren dass Sie Freiwillige suchen zum Thema Allergien im Kindesalter. Ich finde dass Thema selbst recht spannend weil beide Elternteile Allergien haben [...] und ich) und unser [...] (6 Monate) hoffentlich keine davon tragen wird. Was genau müssen wir machen?“ (W4M-LJ37)

Translation: "Hello, I heard from Mrs. [...] that you are looking for volunteers on the subject of allergies in childhood. I find that topic quite exciting myself because both parents have allergies (felix and me) and our [...] (6 months) will hopefully not carry any of them. What exactly do we need to do?"

1. „Hallo grundsätzlich passt 20 Uhr super nur diese Woche hat mein Mann spätdienst und das Risiko ist [...] das mein kleiner Sohn immer mal Wieder wach wird und ich unterbrechen muss... also die woche ab dem 16ten kann ich ab 20 uhr super.“ (W4M-LJ39)

Translation: "Hi, in general 8 pm fits great, but this week my husband is on late duty and the risk is high that my little son will wake up again and again and I have to interrupt... so the week from the 16th I can from 20 o'clock super."

1. „Prinzipiell wären für uns beide Termine ok, wobei der Termin vormittags etwas besser passt, weil da die Kinderbetreuung geregelt ist.“ (SCC-E01 )

Translation: "In general, both dates would be ok for us, whereby the date in the morning fits a bit better, because childcare is arranged then."

1. „gern stelle ich mein Wissen ihrer Studie zur Verfügung. Mein Zeitfenster ist allerdings begrenzt. Eine gute Zeit wäre Dienstags zwischen 8.00 und 9.00.“ (W3M-M08)

Translation: "I would be happy to provide my knowledge to their study. However, my time slot is limited. A good time would be Tuesdays between 8:00 and 9:00."

1. „Ja morgen 11 Uhr (oder die anderen beiden Vormittage auch) würde gehen. Rufen sie mich dann an? Muss ich irgend etwas spezielles vorab raussuchen?“ (W3M-M24)

Translation: "Yes tomorrow 11 am (or the other two mornings as well) would work. Will they call me then? Anything specific I need to pick out in advance?"

1. „Ich habe von der Studie zu Allergien gelesen. Meine Tochter ist 3 Jahre alt und hat keine Allergien.  Wären diese 3 Termine für die Onlinebefragung flexibel?“ (W6M-DI18)

Translation: "I read about the study on allergies. My daughter is 3 years old and has no allergies. Would these 3 dates be flexible for the online survey?”

1. „Gern möchte ich an Ihrer Studie teilnehmen. Könnten Sie mir vorab noch beantworten in welchem zeitlichen Umfang die Befragungen sein werden?“ (W6M-HR10)

Translation: "I would like to participate in your study. Could you answer me in advance in what time frame the surveys will be?"

1. „Eine Frage: sind die Termine zur Befragung fix oder frei wählbar? Da ich berufstätig bin müsste ich diese sonst erst kennen, um zu wissen ob sich das arrangieren lässt.“ (W6M-SE01)

Translation: "One question: are the dates for the survey fixed or freely selectable? Otherwise, since I am working, I would need to know these first to know if this can be arranged."

1. „im August werden wir Eltern und würden beide an ihrer Studie teilnehmen, sofern das voraussichtliche Entbindungsdatum mit dem [...] nicht zu spät für sie ist und die 3 Befragungszeiträume in einem gewissen Zeitfenster flexibel von zu Hause machbar sind. Oder sind dafür Termine notwendig?“ (W6M-WL23)

Translation: "in August we will become parents and would both participate in their study, provided the expected delivery date of [...] is not too late for them and the 3 interview periods can be done flexibly from home in a certain time window. Or are appointments necessary for this?"

1. „Mein Mann und ich würden gerne an der Befragung teilnehmen. Was ist dafür zu tun?“ (W6M-WL24)

Translation: "My husband and I would like to participate in the survey. What needs to be done to make this happen?"

1. „Es wäre mir sehr recht den zweiten Teil vorzuziehen, da um den eigentlich veranschlagten Zeitpunkt in 2 Wochen der Geburtstermin meines Kindes ansteht und es sein kann, dass ich die Sache dann vergesse.“ (W6M-DC78)

Translation: "I would be very comfortable bringing the second part forward, since my child's due date is coming up around the actual scheduled time in 2 weeks and I may forget about it then."

1. „Ich weiß auch nicht wie wir in nächster Zeit dazu kommen da wir aktuell zeitlich sehr eingespannt sind. Ich schau auf jeden Fall das wir abends mal dazu kommen. Gibt es ein Datum bis wann es erledigt sein muss?“ (W6M-HA044)

Translation: "I don't know how we will get to it in the near future because we are currently very busy. I'll definitely see if we can get to it in the evening. Is there a date by when it needs to be done?"

1. „Wann sind denn die Befragungen? Wenn es zeitlich passt könnte mein Mann auch daran teilnehmen“. (W6M-HA013)

Translation: "When are the interviews? If it fits the time, my husband could participate as well."

1. „Leider ist es uns nicht möglich zu diesen beiden Terminen zu kommen, wir haben aber trotzdem Interesse an einer Mitarbeit.“ (SCC-E06)

Translation: "Unfortunately it is not possible for us to come to these two dates, but we are still interested in participating."

**Coping/Managing self-efficacy**

1. „wir könnten spontan am Freitag. Es kann aber sein, dass ich zwischendurch mich zum Stillen zurückziehen werde, da die Kleine noch keinen festen Rhythmus hat.“
   Translation: "We could spontaneously on Friday. However, it may be that I will retire in between to breastfeed, as the little one does not yet have a fixed rhythm." (W4M-LJ40)
2. „Sollte es mir, wegen meinem Baby und dem Kleinkind nicht möglich sein nächste Woche beim Elternbeirat dabei zu sein, konnte ich Ihnen zumindest noch diesen kleinen Einblick von mir als betroffene Mutter zeigen.“ (SCC-E03)
   Translation: "If I am not able to be at the Parents' Council next week because of my baby and toddler, I could at least show you this little insight from me as a concerned mother."
3. „Sehr gerne möchte ich mich an Ihrem Projekt beteiligen. Ich stille jedoch noch voll und bin mir nicht sicher, ob es mit Baby im Oktober machbar ist (aus dem LK Bayreuth). Würde das Baby beim Treffen dabei sein können? Ansonsten wäre ich sehr gerne außerhalb des Treffens dabei.“ (SCC-E03)
   Translation: "I would very much like to participate in your project. However, I am still fully breastfeeding and am not sure if it is feasible with baby in October (from Bayreuth district). Would the baby be able to be at the meeting? Otherwise I would be very happy to be there outside the meeting."
4. „Entschuldigung, ich war viel unterwegs und die E-Mail ist untergegangen. Gern können Sie mich erreichen unter [...]. Heute bin ich gegen 14 und gegen 16 Uhr erreichbar, morgen ab 14 Uhr (wenn ich gerade mit Wickeln o.ä. beschäftigt bin, rufe ich zurück).“ (W4M-LJ53)
   Translation: "Sorry, I've been on the road a lot and the email got lost. You are welcome to reach me [...] Today I am available around 2 p.m. and around 4 p.m., tomorrow from 2 p.m. (if I am busy changing diapers or similar, I will call you back).”
5. „Ich würde gern die Befragung ohne Begleitung durchführen. Ich hoffe, dass ich dazu in der Lage bin.“ (W6M-DC127) "I would like to do the interview unaccompanied. I hope that I am able to do so."
6. „Ich würde den zweiten Teil der Befragung doch ohne Begleitung beantworten - und dies auch gleich zu Beginn der Woche durchführen. Sollte ich merken, dass Rückfragen unerlässlich sind, würde ich mich nochmal melden. Vielleicht wäre dann doch ein Zeitfenster möglich.“ (W6M-HA187)
   Translation: "I would answer the second part of the questionnaire unaccompanied after all - and also do this right at the beginning of the week. If I notice that queries are essential, I would contact you again. Perhaps a time slot would then be possible after all."
7. „Ich hatte schon begonnen, aber musste es dann abbrechen. Zuhause habe ich nur ein Laptop und momentan sehr langsames Internet (2mbit/sek). Werde es nächste Woche ausfüllen.“ (W6M-HA437)
   Translation: “I had already started, but then had to stop. I only have a laptop at home and very slow internet at the moment (2mbit/sec). I will complete it next week."
8. „Ich freue mich sehr über Ihre Mail. Mein Sohn ist 2,5 Jahre alt (Januar [...] geboren) Wir haben alle keine bekannten Allergien. Wenn die Gespräche online stattfinden würden, bin ich offen für Gruppen oder Einzelgespräch.“ (W4M-LJ53)

Translation: "I am very happy to receive your mail. My son is 2.5 years old (born January [...]) We all have no known allergies. If the conversations would be online, I am open to group or one-on-one."

1. „entschuldigen Sie vielmals die Unannehmlichkeit. Aufgrund mehrerer Ereignisse habe ich gestern Abend nicht mehr an die Veranstaltung gedacht und habe sie erst heute morgen wieder im Kalender entdeckt. Findet noch eine Veranstaltung statt? Brauchen Sie noch Teilnehmer? Ich bin gerne bereit noch teilzunehmen. Ich hoffe gestern hat ansonsten alles soweit geklappt!“ (W4M-LJ55)

Translation: "so sorry for the inconvenience. Due to several events, I didn't think about the event last night and just found it again on the calendar this morning. Is there another event taking place? Do you still need participants? I am happy to still participate. I hope everything else worked out so far yesterday!"

1. „es tut mir sehr leid aber ich werde es um 20 Uhr heute doch nicht schaffen! Mein Kleiner ist aus unersichtlichem Grund heute völlig aus dem Schlafrythmus.“ (W4M-LJ57)

Translation: "I'm very sorry but I won't be able to make it at 8pm today after all! My little one is completely out of sleep rhythm today for some unknown reason."

**Resources**

1. „Mein Mann würde entweder nur kurz an dem Gespräch teilnehmen oder gar nicht, da er beruflich stark eingebunden ist. Beste Grüße“ (W4M-LJ42)
   Translation: "My husband would either attend the interview briefly or not at all as he is very busy at work. Best regards"
2. „Am einfachsten wäre eine Teilnahme für mich an einem Montagvormittag, da ich da in der Regel nicht arbeite und mein Kind im Kindergarten ist. Über eine Rückmeldung und weitere Informationen würde ich mich sehr freuen. “ (W4M-LJ43)
   Translation: "It would be easiest for me to participate on a Monday morning, as I don't usually work then and my child is in kindergarten. I would be very pleased to hear from you and receive further information. "
3. „grundsätzlich ist für mich unter der Woche ab 20 Uhr kein Problem. Wenn ich den Termin früh genug >1 Woche erhalte, kann ich mir das so einrichten. Wir sind in der Zeit auch erstmal nicht im Urlaub.“ (W4M-LJ44)
   Translation: "in principle, 8 p.m. is no problem for me during the week. If I get the appointment early enough >1 week, I can arrange it that way. We are not on holiday at that time."
4. „leider haben unsere Hebammen, aufgrund des hohen Arbeitsaufkommens, zur Zeit keine Möglichkeiten an Ihrer Studie teilzunehmen.“ (W3M-M26)
   Translation: "unfortunately, due to the heavy workload, our midwives do not have the opportunity to participate in your study at this time."
5. „Vor einer Woche ist unsere zweite Tochter zur Welt gekommen, so dass ich derzeit im Wochenbett liege. Die neue familiäre Situation macht es mir schwer einzuschätzen, ob ich es zeitlich schaffe. Somit würde ich den Platz freigeben. Falls in den nächsten Monaten/ Jahren erneut solch eine Studie durchgeführt wird, bin Ich auf jeden Fall interessiert. Ich wünsche Ihnen viel Erfolg bei der der Studie und spannende Ergebnisse.“ (W6M-HA405)
   Translation: "A week ago our second daughter was born, so I am currently in postpartum. The new family situation makes it difficult for me to assess whether I can make it in terms of time. Thus, I would release the place. If such a study is conducted again in the next few months/years, I would definitely be interested. I wish you success with the study and exciting results.”
6. „Ich freue mich sehr über Ihre Mail. Mein Sohn ist 2,5 Jahre alt (Januar [...] geboren) Wir haben alle keine bekannten Allergien. Wenn die Gespräche online stattfinden würden, bin ich offen für Gruppen oder Einzelgespräch.“ (W4M-LJ47)
   Translation: "I am very happy to receive your mail. My son is 2.5 years old (born January 2019) We all have no known allergies. If the talk would be online, I am open to group or one to one.”
7. „Es ist jedoch leider noch nicht sicher, ob es mir aus organisatorischen Gründen möglich ist teilzunehmen. Bei einer digitalen Veranstaltung wäre ich jedoch sehr gerne auf jeden Fall dabei.“ (SCC-E03)

Translation: "However, it is unfortunately not yet certain whether it will be possible for me to participate for organizational reasons. However, I would very much like to be there for a digital event in any case."

1. „Gerne dürfen Sie mich bei Bedarf kontaktieren. Digitale Veranstaltungsformate sind im Familienalltag (mit zwei allergischen Kindern) viel einfacher zu integrieren. Über eine Aufnahme in den Informationsverteiler würde ich mich ebenfalls freuen.“ (SCC-E02)
   Translation: "You are welcome to contact me if needed. Digital event formats are much easier to integrate into everyday family life (with two allergic children). I would also be pleased to be included in the information distribution list."
2. „Können wir das Interview gleich jetzt durchführen?” (W3T-P04)
   Translation: “Can we conduct the interview right now?”
3. „Kann ich Sie anrufen, wenn ich mit meinen Patienten fertig bin?" (W3T-P13)
   Translation: “Can I call you once I’m finished with my patients?”
4. „Als mein Freund die erste Befragung gemacht hat gab es Schwierigkeiten. Erst konnte er den Link nicht öffnen und als es endlich klappte und er sich durchgeklickt hatte, ist das Internet abgestürzt. Ich bin mir nicht sicher ob die Daten übermittelt wurden, vielleicht könnten Sie uns den Link nochmals zusenden. (…) Nun hat alles geklappt und mein Freund konnte auch den ersten Teil nochmals ausfüllen. Vielen Dank für den Tipp!“ (W6M-SA33)
   Translation: "When my friend did the first interview there were difficulties. First he couldn't open the link and when it finally worked and he clicked through, the internet crashed. I'm not sure if the data was transmitted, maybe you could send us the link again. (...) Now everything worked and my friend was also able to fill in the first part again. Thank you very much for the tip!"
5. „Frau [...] hat ihre Mail bezüglich der Studie Gesundheit bei Kleinkindern an mich weitergeleitet. Es wäre gut wenn ich diesen Flyer auch auf Arabisch erhalten würde. Gibt es noch weitere Sprachen ? Z.B. [...]? Oder Kurdisch?“ (W4M-LJ41)
   Translation: "[...] has forwarded her mail regarding the study on health in young children to me. It would be good if I could receive this flyer in Arabic as well. Are there any other languages ? E.g [...] Or Kurdish ?"
6. „Also ich lebe seit ca. 10 Jahre in Deutschland, mein Mann 3 Jahre. Wir haben beide Hochschulabschluss. Ich werde in meinem Umfeld anschauen ob es mir jemand einfällt.. Vielleicht ein Tipp, ich habe lange bei Flüchtlingskinder gearbeitet, beim solche Organizationen können Sie solche Eltern ganz einfach finden. Aber meist sprechen sie kaum Deutsch. Tut mir Leid dass ich sie in diesem Fall nicht unterstützen kann. Wenn etwas anderes gibt, sind wir gerne bereit Ihnen zu unterstützen.“ (W4M-LJ64)
   Translation: “Well, I've been living in Germany for about 10 years, my husband for 3 years. We both have university degrees. I will look in my environment to see if I can think of someone... Maybe a tip, I have worked with refugee children for a long time, at such organisations you can easily find such parents. But mostly they hardly speak German. I'm sorry that I can't support you in this case. If there is something else, we are happy to support you."
7. „mein Mann hat heute auch eine zusätzliche Telko beruflich rein bekommen. Ich versuche trotzdem an dem Online Gespräch teilzunehmen. Falls dies aufgrund von Kinderbetreuung nicht möglich sein sollte oder ich unterbrechen muss, nehme ich gern auch am nächsten Termin erneut teil. Leider sind Termine mit einem 3 Monate altem Kind zum Teil schwierig zu planen.“ (W4M-LJ45)

Translation: "my husband also got an additional telco in today professionally. I will still try to participate in the online conversation. If this is not possible due to childcare or if I have to interrupt, I will be happy to participate again at the next appointment. Unfortunately, appointments are sometimes difficult to schedule with a 3-month-old child."

1. „Am 7.7. Um 16.00 würde gut passen. Ich hoffe Video geht, aber zur Sicherheit meine Nummer [...]“ (W4M-LJ46)

Translation: "On 7/7 at 4pm would be a good fit. I hope video goes, but just to be safe my number [...] "

1. „Ich konnte ehrlich gesagt, mir die Seiten nicht wirklich angucken, weil mein Kind heute gar nicht mehr mag.“ (W4I-FG7)

Translation: "I honestly couldn't really look at the sites because my kid doesn't like today at all.”

1. „Zu den Terminen Ende Oktober kann ich allerdings nicht vor Ort sein. Eine Besprechung via Videotelefonie kann ich meistens auch kurzfristig organisieren.“ (SCC-E02)

Translation: "However, I can't be on site for the appointments at the end of October. I can usually arrange a meeting via video phone on short notice."

1. „Ich kam bisher noch nicht dazu. Nun wollte ich beginnen, nun sind heute Wartungsarbeiten im Gange...., ich probiere es ein andernmal wieder.“ (W6M-DC85)

Translation: "I haven't gotten around to it yet. Now I was going to start, now there is maintenance going on today...., I'll try again another time."

1. „Frau [...] hatte ich vor Beginn des 1. Teils mitgeteilt, dass mein Mann wegen Zeitmangel leider doch nicht teilnehmen kann.“ (W6M-SA58)

Translation: "I had informed Mrs. [...] before the start of the 1st part that unfortunately my husband would not be able to participate after all due to lack of time."

1. „Vielen lieben Dank für die Erinnerung an den zweiten Teil. Mit Neugeborenem ist man ziemlich eingespannt und vergisst Dinge schnell. Ich habe den zweiten Teil soeben bearbeitet.“ (W6M-HA401)

Translation: "Thank you very much for reminding me about the second part. With a newborn you are pretty plugged in and forget things quickly. I have just finished the second part."

**Support**

1. „ja ich hatte mir das Gespräch auch notiert aber die letzten Tage waren etwas turbulent da unser Sohn erhöhte Temperatur hatte. Kann ich mich heute im Laufe des Tages bei Ihnen telefonisch melden? Aktuell schläft unser Sohn gerade neben mir und ich würde gerne noch bei ihm bleiben.“ (W4M-LJ50)
   Translation: "Yes, I had made a note of the conversation but the last few days have been a bit turbulent as our son has had a high temperature. Can I call you today during the course of the day? Currently our son is sleeping next to me and I would like to stay with him."
2. „aktuell habe ich wirklich sehr viel zu tun. Unter der Woche schaffe ich es nicht. Könnten wir evtl. am Sa Vormittag telefonieren? Rufen sie mich bitte am Handy einfach an.“ (W3M-P21)
   Translation: "I'm really busy at the moment. I can't make it during the week. Could we possibly talk on the phone on Saturday morning? Just call me on my mobile, please."
3. „Wir würden den link ohne Hilfe nehmen, dann können wir spontan entscheiden, wann wir die Umfrage machen.“ (W6M-HA123)

Translation: "We would take the link without help, then we can decide spontaneously when to do the survey."

1. „leider konnte ich noch keine Buchung auf meinem Konto sehen. Ich bitte um Prüfung.“ (W4M-LJ51)

Translation: "unfortunately I could not see any posting on my account yet. I ask for verification."

1. „Ich wollte mal kurz nachfragen, den Brief mit meiner Bankverbindung habe ich schon vor ein paar Wochen zurück geschickt. Ich habe aber bisher noch nichts überwiesen bekommen. Wann wird das denn gemacht?“(W4M-LJ52)

Translation: "I just wanted to check, I already sent back the letter with my bank details a few weeks ago. But I haven't gotten anything transferred yet. When will that be done?"

1. „Vielen Dank für die Erinnerungen. Diese brauchen wir!“ (W6M-SA114)
   Translation: "Thank you for the reminder. We need these!"
2. „Ohne den Fragebogen 2 zu kennen, würde ich einmal sagen, dass ich (oder auch wir) ihn ohne Videobegleitung ausfüllen kann. Oder zumindest zunächst versuchen werde. Sie können mir sehr gerne die Lösungsblätter zukommen lassen. Ich bin bei solchen Dingen neugierig. Vielen Dank für Ihre Hilfe.“ (W6M-HA369)
   Translation: "Without knowing questionnaire 2, I would say that I (or we) can fill it out without video accompaniment. Or at least will try at first. You are very welcome to send me the solution. I am curious about such things. Thank you very much for your help."
3. „ich möchte mich ganz herzlich bei Ihnen bedanken, dass Sie sich die Zeit genommen haben mir zu antworten. Ich schätze dies sehr. Vielen Dank auch, dass Sie sich diesem Thema in Ihrer Forschungsarbeit angenommen haben. Ich hoffe, dass dies in Zukunft für andere Eltern die wichtige Aufgabe der Allergieprävention ihrer Kinder vereinfacht.“ (SCC-E03)
   Translation: "I would like to thank you very much for taking the time to reply to me. I appreciate this very much. Thank you also for addressing this issue in your research. I hope this will make the important task of allergy prevention for their children easier for other parents in the future."
4. „Ich möchte den Fragebogen erstmal ohne Begleitung ausfüllen. Falls ich feststelle, dass es überhaupt nicht funktioniert, melde ich mich dann nochmal.“ (W6M-DC34)

Translation: "I would like to fill out the questionnaire unaccompanied for now. If I find that it doesn't work at all, I'll get back to you then."

1. „Wir würden den Fragebogen gerne ohne Zoom-Begleitung ausfüllen.“ (W6M-DC54)

Translation: "We would like to fill out the questionnaire without Zoom accompaniment.”

1. „Ich benötige keine Begleitung bei der Bearbeitung. Vielen Dank aber für das Angebot.“ (W6M-DC69)

Translation: "I don't need any accompaniment during the processing. Thank you for the offer though."

1. „Sollte es Probleme geben kann ich mich ja melden und ggf bei Befragung 3 eine Begleitung hinzuziehen.“ (W6M-DC92)

Translation: "If there are any problems I can get in touch and have a companion if necessary for interview 3."

1. „Sind die Fragen denn so ähnlich wie im ersten Teil? Dann sollte ich eigentlich keine Begleitung benötigen, denke ich, denn bislang hatte ich keine Rückfragen. Könnte ich denn ansonstem im Notfall ohne Begleitung anfangen und dann doch abbrechen und auf den
   "begleiteten Weg" umschwenken, falls ich alleine gar nicht zurecht komme?“ (W6M-DC133)

Translation: "Are the questions similar to the first part? Then I shouldn't really need an escort, I think, because so far I haven't had any queries. In case of emergency, could I start without guidance and then break off and switch to the "guided path"?

"accompanied path" if I can't manage on my own at all?"

1. „bitte schicken Sie erneut den link für mich Frau [...], da ist etwas schief gelaufen, der link ist nicht vollständig und kann deshalb nicht angeklickt werden.“ (W6M-DC170)

Translation: "please resend the link for me Ms. Prater, something went wrong, the link is not complete and therefore cannot be clicked."

1. „Mein Mann und ich wollen gern noch an der Umfrage teilnehmen. Gern würde ich diese Woche an einem Zoom Meeting teilnehmen. Zeitlich würde ich hier ab 18:30 Uhr bevorzugen. Wie würde es da bei Ihnen gehen?“ (W6M-DC199)

Translation: "My husband and I would still like to participate in the survey. I would be happy to attend a Zoom meeting this week. Time-wise, I would prefer here starting at 6:30 pm. How would that work for you?"

1. „Oh, nein, ich habe den 3. Teil noch nicht gemacht. Danke, für die Erinnerung, ich habe es tatsächlich total vergessen 🙈🙈🙈🙈 Schande über mein Haupt. Ich werde den Teil ganz bequem heute Abend machen, wenn das Kind schläft.“ (W6M-DC253)

Translation: "Oh, no, I haven't done the 3rd part yet. Thanks, for reminding me, I actually totally forgot 🙈🙈🙈🙈 Shame on me. I will do that part quite comfortably tonight when the child is asleep."

1. „Vielen Dank für die Unterstützung über Zoom. Da ich aber noch ein Baby habe und die Betreuung tagsüber nur über mich erfolgt , mache ich die Befragung dann doch ohne Hilfe.“ (W6M-SA47)

Translation: "Thank you very much for the support via Zoom. But since I still have a baby and the care is only about me during the day , I'll do the survey without help after all."

1. „Beim ausfüllen des ersten Fragebogens hatten wir keine Probleme, ich denke dass es beim nächsten also auch nicht unbedingt erforderlich wäre ein Coaching zu haben. Da mein Mann und ich diesen nicht exakt zur gleichen Zeit ausfüllen, ist es für uns auch einfacher wenn wir flexibel bleiben können.“ (W6M-SA72)

Translation: "When filling out the first questionnaire we had no problems, so I think that with the next one it would not be necessary to have coaching. Since my husband and I don't fill this out at exactly the same time, it's also easier for us if we can stay flexible."

1. „Danke für die Erinnerung. Ich wollte mich eigentlich nach der letzten Email melden, aber bei uns war vor unserem Urlaub so viel zu erledigen, dass ich es einfach zeitlich nicht
   mehr geschafft hatte. Kaum ist man dann im Urlaub, vergisst man einiges und daher bin ich froh für die Erinnerung.“ (W6M-SA92)

Translation: "Thank you for the reminder. I was going to check in after the last email, but we had so much to do before our vacation that I just couldn't make it in terms of time

I just didn't have the time. Then as soon as you are on vacation you forget some things so I am glad for the reminder."

1. „Wird eine Begleitung angeboten, um die Fragen zu verstehen? Also gibt es da z. B. Fachbegriffe?“ (W6M-HA331)

Translation: "Is there any guidance offered to understand the questions? So, for example, are there technical terms?"

1. „Ich habe heute Abend mit der 2. Befragung begonnen. Leider kam ich nach der ersten "Zeitfrage" (Grafik Körpergewicht Perzentile) nicht weiter, hatte nur noch einen weißen Bildschirm. Auch bei jedem weiteren Neustart Versuch kam ich nicht mehr in die Umfrage. Kann es sein, dass ich mich gesperrt habe? Werde es morgen Abend nochmals probieren. Vielleicht haben Sie eine Idee, woran es liegt?“ (W6M-HA237) >support

Translation: "I started the second interview tonight. Unfortunately, after the first "time question" (graph body weight percentile) I did not get any further, just had a white screen. I also did not get into the survey on each subsequent restart attempt. Could it be that I have locked myself out? Will try again tomorrow evening. Maybe you have an idea what the problem is?"

1. „Meine Frau hat die Befragung heute durch geführt, jetzt wollte ich das auch machen, kann ich aber leider nicht, da ich die Meldung bekomme ich hätte die Befragung bereits gemacht. Ich kann mir vorstellen das diese Meldung kommt da ich die Befragung vom selben Rechner durch führe wie meine Frau. Gibt es da eine Lösung für?“ (W6M-HA312)

Translation: "My wife did the survey today, now I wanted to do the same, but unfortunately I can't, because I get the message that I already did the survey. I can imagine that this message comes because I run the survey from the same computer as my wife. Is there a solution for this?"

**Action**

**Formative experience assessment**

1. „anbei das Formular Auszahlung der Aufwandsentschädigung. Das Online Gespräch hat mir sehr gefallen. Ihre Kollegin hat das super gemacht.“ (W4M-LJ58)
   Translation: "Enclosed is the form for payment of the expense allowance. I really enjoyed the online interview. Your colleague did a great job."
2. „Nochmals vielen Dank für das nette und zielführende Gespräch. Anbei mein Code: XX Viel Erfolg für Ihre Studie!“ (W4M-LJ59)
   Translation: "Thank you again for the nice and purposeful conversation. Enclosed is my code: XX Good luck for your study!"
3. “P2: There is actually nothing else. The most important thing is that you reached the goal – by interviewing us, and I wish you good luck in doing your research.
   I: Thank you. (mentioned name), would you like to add something before I stop recording? P2: Nothing, thank you. We talked a lot, and we shared useful things. I hope you reached what you wanted through this interview. And I wish you all success.” (W4I-AR5)
4. „Ich fand den ersten Teil ehrlich gesagt schwieriger, weil man da viele Einschätzungen auf einer Skala 1-6 abgeben musste zu Fragen, die eher vage formuliert waren, während man hier eher Fragen nach dem Ja/Nein-Schema hatte. Ich habe auch meinen Mann nochmal angespitzt, den Fragebogen zu bearbeiten.“ (W6M-DC290)
   Translation: "To be honest, I found the first part more difficult because you had to give a lot of assessments on a scale of 1-6 to questions that were formulated rather vaguely, whereas here you had more yes/no questions. I also pushed my husband again to do the questionnaire."

1. „Es hat alles gut geklappt, ich war nur etwas überrascht über die vielen mathematischen Fragen. Ich hoffe ich habe richtig gerechnet.“ (W6M-SA26)

Translation: "It all worked out well, I was just a bit surprised by all the maths questions. I hope I did the maths right."

1. „Alles in alle fand ich die Zweite am besten und die Dritte am schlechtesten. Ich dachte hier ging es mehr bzgl über Allergien bei Kindern mit Augenmerk unter 3 Jahren... die letzte Umfrage mit Corona hatte für mich nichts damit zu tun. Ob ich das alles so richtig beantwortet habe, weiss ich auch nicht.“ (W6M-KW32_03)
   Translation: "All in all, I found the second best and the third worst. I thought this was more about allergies in children under the age of 3... the last survey with Covid had nothing to do with that for me. I don't know if I answered all the questions correctly.”
2. „Ich muss sagen die Wahrscheinlichkeitsfrage fand ich etwas enttäuschend. (Wahrscheinlichkeiten dass Jungen/Mädchen die Allergie xy haben). Ich finde die Wahrscheinlichkeiten hätten so gewählt sein müssen dass wenn mans aufaddiert man auf über 20% kommt, wenn man allerdings die Wahrscheinlichkeiten für eines der drei ausrechnet man auf unter 20 kommt (wenn wir von unabhängigen Variablen ausgehen)e (hoffentlich habe ich mich da nicht verlesen/verrechnet sonst ist die Kritik peinlich =) ) (und ja mir ist klar dass es wahrscheinlich nicht darum geht herauszufinden ob jemand Wahrscheinlichkeitsrechnung beherrscht...).“ (WP6-HA203)
   Translation: "I have to say that I found the probability question a bit disappointing. (Probabilities that boys/girls have allergy xy). I think the probabilities should have been chosen in such a way that if you add it up you get over 20%, but if you calculate the probabilities for one of the three you get under 20 (if we assume independent variables)e (hopefully I didn't read it wrong/ miscalculate or else the criticism is embarrassing =) ) (and yes I realise that it's probably not about finding out if someone knows probability...)."
3. „Vielen Dank für die Links, ich finde sie beide sehr hilfreich. Ich habe direkt das abgespeichert und werde danach mehr lesen.“ (W4I-FG11)

Translation: "Thank you for the links, I find them both very helpful. I directly saved the one and will read more after that."

1. „Deswegen vielleicht, also wir kommen bestimmt noch drauf, aber ich war vorhin total begeistert, als ich diese Links von Ihnen aufgemacht habe, weil ich mir gedacht habe, das ist ja genau das, was ich in der Schwangerschaft schon gesucht habe. Und ich weiß nicht, also warum ich da nicht drauf gestoßen bin, aber genau, kommt wahrscheinlich noch. B: (unv.), ach super. Nein, aber freut mich schon, weil alleine dadurch haben Sie mir schon ein Stück weit weitergeholfen. Weil ich jetzt dann zwei Anlaufstellen habe und zumindest von dem AAK finde ich sehr, sehr schön übersichtlich gestaltet. Also ich glaube, da ist inhaltlich richtig gut was für mich dabei.“ (W4I-E14)

Translation: "That's why maybe, so we'll definitely get to it, but I was totally excited earlier when I opened these links of yours because I thought to myself, this is exactly what I was already looking for in pregnancy. And I don't know, so why I didn't come across it, but exactly, probably coming. B: (unv.), oh great. No, but I'm glad, because that alone has helped me a bit. Because now I have two contact points and at least from the AAK I find very, very nicely clearly designed. So I think there's something really good for me in terms of content."

1. „auch ich fand unser Gespräch sehr nett! Es hat für mich den Stellenwert der Allergieprävention nochmal in den Vordergrund bei meiner täglichen Arbeit gerückt, das ist Klasse. Anbei übersende ich Ihnen die Teilnahmebescheinigung sowie die Einwilligung zur Studienteilnahme. Herzliche Grüße und weiter viel Erfolg,“ (W3M-P14)

Translation: "I also found our conversation very nice! For me, it has once again brought the importance of allergy prevention to the fore in my daily work, which is great. Enclosed I am sending you the certificate of participation as well as the consent to participate in the study. Best regards and continued success,"

1. „das Interview hat auch mir viel Spaß gemacht und tatsächlich überlege ich, wie ich das Thema Allergien /Allergieprophylaxe noch deutlicher in meine Anamnesen, bzw. Aufklärungsgespräche einbinden könnte. Sehr wertvoll, Ihre Studie! Im Anhang sende ich Ihnen den Scan von der Teilnahmebescheinigung.“ (W3M-M25)

Translation: "the interview was a lot of fun for me, too, and in fact I am thinking about how I could incorporate the topic of allergies /allergy prophylaxis even more clearly into my case histories, or educational discussions. Very valuable, your study! I am sending you the scan from the certificate of participation in the attachment."

1. „Ich möchte die zweite Befragung gerne ohne Beratung erledigen, da ich immer mal wieder daran arbeite und es wahrscheinlich nicht an einem Stück schaffe. Die erste war erträglich, zum Glück wusste ich noch einigermaßen wie Prozentrechnung funktioniert.“ (W6M-DC104)

Translation: "I'd like to get the second survey done without counseling, since I'm working on it on and off and probably won't be able to do it in one piece. The first one was tolerable, luckily I still knew how percentages worked to some extent."

1. „Hatte mich über die vielen Statistik Fragen gewundert, aber bin gut zurecht gekommen. Hab also den ersten Fragebogen ausgefüllt.“ (W6M-DC140)

Translation: "Had wondered about all the statistics questions, but did okay. So filled out the first questionnaire."

1. „Ich finde die Umfrage übrigens wirklich informativ und hilfreich; haben Sie
   zufällig eine Zusammenfassung der vielen Informationen, die Sie dort (sehr gut) präsentieren?“ (W6M-DC158)

Translation: "By the way, I find the survey really informative and helpful; did you

a summary by any chance of all the information you present there (very well)?"

1. „Wenn der Fragebogen ähnlich wie der erste ist, brauchen wir keine Begleitung. Oder gibt es diesmal "besondere Hausforderungen?“ (W6M-DC160)

Translation: "If the questionnaire is similar to the first one, we don't need an escort. Or are there any "special house calls" this time?"

1. „Die Fragen sind nicht schwer zu beantworten, nur manchmal etwas kompliziert gestellt.“ (W6M-SA96)

Translation: "The questions are not difficult to answer, just sometimes asked in a complicated way."

1. „Mit der dritten Befragung bin ich fertig. Mein Mann möchte an der Befragung doch nicht teilnehmen- es dauert ihn zu lange.“ (W6M-SA101)

Translation: "I am done with the third interview. My husband doesn't want to participate in the survey after all- it takes him too long."

1. „Wir haben beide Teile ausgefüllt. Es ist immer wieder sehr interessant.“ (W6M-SA107)

Translation: "We have completed both parts. It's always very interesting."

1. „Bin auf die Ergebnisse der Studie gespannt und hoffe, dass ihr gute Erkenntnisse erzielt. Eine Frage habe ich dennoch. Ihr fragt danach, ob mein Kind eine Allergie hat. Hier ist lediglich die Auswahl ja oder nein möglich. Was ist aber mit Probanden, die es nicht wissen?
   Den Antworten zufolge, könnt ihr uns lediglich in zwei Zielgruppen einordnen:

- Eltern, deren Kind eine Allergie hat

- Eltern, deren Kind keine Allergie hat

Wäre es für euch nicht interessant zu erfahren und eine Zielgruppe zu bilden mit Eltern, die nicht wissen, ob ihr Kind eine Allergie hat? Ich zum Beispiel weiß es gerade nicht und werde fälschlicherweise in die „falsche“ Zielgruppe eingeordnet, wenn ihr Auswertungen anhand dieser Kriterien fahren würdet.“ (W6M-HA82)

Translation: "I'm looking forward to the results of the study, and I hope you guys come up with some good findings. I do have one question though. You ask about whether my child has an allergy. Here you can only select yes or no. But what about participants who don't know?

According to the answers, you can only classify us into two target groups:

- Parents whose child has an allergy

- Parents whose child does not have an allergy

Wouldn't it be interesting for you to know and form a target group with parents who don't know if their child has an allergy? I, for example, don't know right now and would be mistakenly placed in the "wrong" target group if you ran evaluations based on these criteria."

1. „Hab grad den ersten Teil beantwortet. Ist ja ganz schön trickie. Bei den Textaufgaben hatte ich auch ein paar mal Kopfzerbrechen. 😉 Bin auf den zweiten Teil gespannt.“ (W6M-HA301)

Translation: "Just answered the first part. It's quite tricky. I also had a few headaches with the text tasks 😉 I'm looking forward to the second part."

1. „Hab grad Teil zwei gemacht. Der war ja deutlich entspannter als Teil 1.“ (W6M-HA338)

Translation: "I just did part two. That one was a lot more relaxed than part one."

1. „Bekommen die Studienteilnehmer eine Kopie der Ergebnisse /Einsicht in die gezogenen Schlüsse? (Mich würde wirklich interessieren, was mathematisches Grundwissen und das Verständnis von Textaufgaben mit dem Risiko für Kinder, eine Allergie zu entwickeln, zu tun hat.“ (W6M-HA350)

Translation: "Do study participants get a copy of the results/insight into the conclusions drawn? (I'd be really interested in what basic math knowledge and understanding of text problems has to do with children's risk of developing allergies."

1. „Für dich zur Info - Den ersten Fragebogen habe ich ausgefüllt. Bin gespannt auf den nächsten in 3-4 Wochen.“ (W6M-HA047)

Translation: "For your info - I have filled out the first questionnaire. Looking forward to the next one in 3-4 weeks."

**Maintenance motivation**

1. „Mir ist jetzt nur durch die ganze Teilnahme an der Studie auch aufgefallen, wie wenig Informationen man eigentlich kriegt zu Allergien, wenn man nicht gerade betroffen ist.“ (W4I-FG1)
   Translation: "I just noticed through the whole participation in the study how little information you actually get about allergies if you are not actually affected."
2. „Für mich wäre es jetzt wichtig, nach unserem Gespräch vor allem auch noch mal, zu wissen, was kann ich selbst aktiv dafür tun. Also in Bezug auf meine Ernährung vielleicht schon in der Schwangerschaft, was in der Stillzeit die Ernährung, aber auch wie führe ich mein Kind an Lebensmittel ran, welche sind da ganz gut, um es zu vermeiden. Keine Ahnung, wie geht man im Kindergarten damit um. Also da noch mal auf die Ernährung achten. Also ich muss sagen, jetzt nach dem Gespräch, ich werde mich auf jeden Fall noch auf die Suche nach Informationen machen nach hochwertigen.“ (W4I-E18)
   Translation: "For me it would now be important, after our discussion, to know again what I can actively do myself. So in terms of my diet, maybe already during pregnancy, what about breastfeeding, but also how do I introduce my child to foods, which ones are quite good to avoid. I don't know how to deal with it in kindergarten. So I have to pay attention to nutrition again. So I have to say, now that we've talked, I will definitely look for more information about high-quality foods.”
3. „Bin wirklich gespannt wie die Ergebnisse ausfallen werden. Generell noch eine Frage zum 3. Teil. War der Corona-Teil von Anfang an Teil dieser Studie oder habt ihr das nachträglich abgeändert? Ich frage deswegen nach, weil ich in diesem Teil keinen Bezug zum eigentlichen Thema „frühkindliche Allergieprävention“ gesehen habe. Wie passen die beiden Themen denn zusammen?“ (W6M-HA281)
   Translation: "I'm really curious to see what the results will be. In general, one more question about the third part. Was the Corona part part of this study from the beginning or did you change it afterwards? I'm asking because I didn't see any reference to the actual topic of "early childhood allergy prevention" in this part. How do the two topics fit together?
4. „Grundsätzlich ist es mir bereits nach der ersten Befragung, schwer gefallen, mich für die kommende zu motivieren. Das Gefühl wurde stärker bei der zweiten Befragung. Der Bezug zum Thema fehlte, ich erkannte keinen Sinn hinter dem, was ich machte. Stattdessen fühlte es sich nach einer Abfrage meiner Intelligenz an. Das teilte ich Ihrer Vorgängerin mit, leider ohne Rückmeldung. Mit dem letzten Funken Hoffnung, startete ich die letzte Befragung Anfang der Woche und habe sie dann abgebrochen. Ich bin wirklich verärgert. Wie das Vervollständigen von Sätzen und die Corona Pandemie, zukünftig Eltern zum Thema Allergien helfen soll, ist mir schleierhaft. Dieses Anliegen wollte ich sehr gerne unterstützen und nichts anderes. Dafür ist mir meine Zeit zu kostbar.“ (W6M-HA416)
   Translation: "Basically, after the first interview I already found it difficult to motivate myself for the next one. The feeling became stronger during the second interview. The connection to the topic was missing, I didn't see any sense behind what I was doing. Instead, it felt like a quiz on my intelligence. I communicated this to your predecessor, unfortunately without any feedback. With the last shred of hope, I started the final interview earlier this week and then abandoned it. I am really annoyed. How completing sentences and the Covid Pandemic is supposed to help parents in the future on the subject of allergies is beyond me. I wanted very much to support this cause and nothing else. My time is too precious for that."
5. „Es hängt zurzeit an meinem Mann, der den ersten Fragebogen noch nicht ausgefüllt hat. Wir sind für eine Woche in Urlaub gefahren und holen die Befragung nächste Woche direkt nach.“ (W6M-DC71)

Translation: "It's currently up to my husband, who hasn't filled out the first questionnaire yet. We went on vacation for a week and we'll get to the survey next week."

1. „Ich habe die erste Befragung gerade abgeschlossen. Vielleicht hätten sie
   die Leute vorwarnen sollen, dass es so viele Matheaufgaben in der Umfrage
   gibt.“ (W6M-DC81)

Translation: "I just finished the first interview. Maybe they should have warned people that there were so many math tasks in the survey."

1. „Leider haben wir es noch nicht geschafft mit dem zweiten Teil der Fragebogen. Hab vor 2 Wochen entbunden und ist ganz viel mit alles drum und dran. Sobald ich die Zeit finde, melde ich mich bei Ihnen.“ (W6M-SA130)

Translation: Unfortunately, we have not yet made it with the second part of the questionnaire. I gave birth 2 weeks ago and it’s quite busy with everything. As soon as I find the time, I'll get back to you."

1. „Ich muss aber gestehen, die Matheaufgaben habe ich nicht erwartet. Und mein Kopf war auch nicht wirklich fit dafür. Kommen davon noch viele, in den anderen Befragungen? Ich hoffe nicht (bitte).“ (W6M-HA381)

Translation: "I have to admit, though, I wasn't expecting the math questions. And my head wasn't really fit for it either. Are there many more of these coming, in the other interviews? I hope not (please)."

1. „Eine wirklich sehr interessante Befragung! Werden bei der Präsentation im Herbst auch die richtigen Antworten mitgeteilt? Es ist ja doch spannend zu erfahren, ob man das richtige angekreuzt hat 🙂 Ich hoffe, dass Sie eine aussagekräftige Studienaussage erhalten.“ (W6M-392)

Translation: "A very interesting survey! Will the correct answers also be shared at the presentation in fall? After all, it's exciting to know if you checked the right one 🙂 I hope you get meaningful study results."

1. „Leider könnte ich in [...] nicht dabei sein, freue mich aber, wenn ich dennoch berücksichtigt werden kann oder anderweitig zur Studie beitragen kann.“ (SCC-E10)

Translation: Unfortunately, I would not be able to be in [...], but will be happy if I can still be considered or otherwise contribute to the study."

**Continuing decision**

1. „anbei das unterschriebene Formular zur Rückerstattung. Vielen Dank für den tollen Austausch, wir nehmen sehr gerne weiterhin an solchen Terminen teil.“ (W4M-LJ60) Translation: "enclosed is the signed form for reimbursement. Thank you very much for the great exchange, we will be very happy to continue participating in such appointments."
2. „Mein Partner, wie auch ich haben den ersten Teil der Befragung bereits vor
   Wochen ausgefüllt. Das Ausfüllen des Fragebogens ist uns beiden leicht gefallen, gerne würden wir weiter an der Studie teilnehmen!“ (W6M-DC280)
   Translation: "My partner and I already filled out the first part of the survey weeks ago. It was easy for both of us to fill in the questionnaire, we would like to continue participating in the study!"
3. „Ansonsten wollte ich Ihnen noch sagen dass mir das Gespräch viel Spaß gemacht hat sollten Sie oder Ihre Kolleg:innen noch für andere Studien Teilnehmer:innen suchen können Sie mir gerne Bescheid sagen!“ (W4M-LJ62)
   Translation: "Apart from that, I wanted to tell you that I enjoyed the interview very much. If you or your colleagues are still looking for participants for other studies, please let me know!
4. „Meinen Mann frage ich gleich noch mal, aber auf Grund der momentan etwas
   knappen Zeit würde ich vermuten, dass er es eher nicht schafft.“ (W6M-DC115)
   Translation: "I'll ask my husband again in a moment, but due to the shortage of time at the moment. I'm guessing he won't be able to make it."

1. „Bitte entschuldigen Sie, mir ist die Studie leider entfallen. Aus Zeitgründen kann ich leider doch nicht daran teilnehmen.“ (W6M-SA46)
   Translation: "Please excuse me, unfortunately the study slipped my mind. Due to time constraints, I'm afraid I can't take part after all."
2. „Ich habe versucht ihre Fragen wahrheitsgemäß zu beantworten, doch zum Ende hin habe Ihre Befragung abgebrochen. Bitte verlangen Sie nicht von einer Mutter mit Baby, die gegen 23Uhr Zeit für die Befragung findet (!), sich noch mit Prozent- und Wahrscheinlichkeitsrechnung zu befassen. Diese Fragen haben für mich auch keinen Sinn mehr gemacht, außer Sie wollen die mathematischen Kenntnisse der Studienteilnehmer abfragen oder falsche Antworten von denen, die auf jeden Fall das Geld möchten. Ich hoffe, Sie können mit meinem Feedback etwas anfangen.“ (W6M-HA299)
   Translation: “Your questioning has been cancelled. Please don't ask a mother with a baby who finds time for the survey at 11pm (!) to deal with percentages and probability calculations. These questions no longer made sense to me, unless you want to test the mathematical knowledge of the study participants or get wrong answers from those who definitely want the money. I hope you can do something with my feedback."
3. „Teil 3 hat uns sehr geärgert auszufüllen, denn wir finden das hat wenig bis nichts mit der Studie zu tun! Unsere Kinder haben beide Zöliakien und ich selbst auch, jeweils sehr einschränkend, das ist der Grund gewesen, weshalb wir teilnehmen wollten. Ebenso haben mein Mann und ich Heuschnupfen. Eine Corona Infektion hat für uns nichts mit einem Allergierisiko unserer Kinder zu tun! Hätten wir vorher gewusst, dass wir über Corona ausgefragt werden, hätten wir nicht teilgenommen. Sehr schade.“ (W6M-DC312)

Translation: "Part 3 was very annoying for us to fill out, because we think it has little to nothing to do with the study! Our children both have celiac disease and so do I, each very limiting, that has been the reason we wanted to participate. Likewise, my husband and I have hay fever. For us, a Covid infection has nothing to do with an allergy risk for our children. If we had known beforehand that we would be quizzed about Corona, we would not have participated. Very unfortunate."

1. „Ich habe ja schon vor einigen Wochen die zweite Umfrage ausgefüllt. Mein Mann ist allerdings so beschäftigt, dass er im Moment nicht dazu kommt, leider. Er möchte es ja auch ordentlich machen. Wäre es eine Option, dass doch nur ich den dritten Fragebogen ausfülle und er die Teilnahme abbricht bzw. zeitversetzt irgendwann ausfüllt wenn er dazu kommt? Ich kann aber nicht sagen wann es soweit wäre, und ich würde gern schon weiter machen mit dem dritten Teil, wenn dies möglich wäre.“ (W6M-SA97)

Translation: "I did fill out the second survey a few weeks ago. However, my husband is so busy that he can't get to it at the moment, unfortunately. He wants to do it properly. Would it be an option that only I fill out the third questionnaire and he cancels the participation or completes it at a later date? But I can't say when it would be the right time, and I would like to continue with the third part already, if this would be possible."

1. „Da meine Tochter bereits im Dezember 3 Jahre wird und ich keine Zeit für Projekttreffen mit Reiseaktivität habe, komme ich als Elternbeirat nicht in Frage.“ (SCC-E07)

Translation: “Since my daughter is already turning 3 in December and I don't have time for project meetings with a travel activity, I'm not eligible to be a parent council member."

1. „Hallo, Das ist keine Studie, das ist eine Prüfung. Ich mache es nicht mehr, danke aber trotzdem. Es hat wirklich viel Zeit gedauert, und ich komme langsam weiter.“ (W6M-HA441)

Translation: "Hello, This is not a study, this is a test. I don't do it anymore, but thank you anyway. It really took a lot of time, and I'm getting there."

**Summative assessment**

1. „Ja, die Teilnahme hat mir Spaß gemacht! Die Studie selbst hat ja auch schon einen großen Informationsgehalt für alle Eltern, das ist sicherlich auch so gewollt. Ich habe sie schon weiterempfohlen und werde das auch nochmals tun! Vielen Dank auch für alle weiteren Informationen dazu.“ (W6M-SA106)
   Translation: "Yes, I enjoyed participating! The study itself already has a lot of information content for all parents, which is certainly the intention. I have already recommended it to others and will do so again! Thank you very much for all the additional information.
2. „vielen Dank nochmal, dass ich an ihrer Studie teilnehmen durfte, ich persönlich fand es sehr spannend und bin auf das Endergebnis gespannt. Ich wünsche ihnen weiterhin noch viel Erfolg! Anbei schicke ich ihnen das ausgefüllte Formular.” (W4M-LJ61)

Translation: "Thank you again for letting me take part, I personally found it very exciting and am looking forward to the final result. I wish them continued success! Enclosed I send them the completed form."

1. „Ich habe ihn eben ausgefüllt. Sehr interessante Fragen. Super.“ (W6M-HA370)

Translation: „I just filled it in. Quite interesting questions. Great.”

1. „War sehr interessant. Konnte einiges neues dazu lernen.“ (W6M-HA425)

Translation: „Quite interesting. Have learned quite something new.”

1. „Danke das ich an dieser Interessanten Umfrage Teilnehmen durfte“. W6M-KW42_13)

Translation: „Thanks for letting me take part in this interesting survey.”

1. „Vielen Dank für den interessanten Fragebogen. Hat Spaß gemacht!“ (W6M-KW39_2)

Translation: „Many thanks for that interesting survey. It was fun!”

1. „Ich bedanke mich für die unkomplizierte Kommunikation und wünsche Ihnen in Ihrer Arbeit weiterhin viel Erfolg.“ (W6M-KW39_8)

Translation: „Thank you for this uncomplicated communication and I wish you success for your further work.”

1. „Für uns beide war es sehr interessant, wir haben uns gerne die Zeit dafür genommen.“ (W6M-KW39_32)

Translation: „It was quite interesting for both of us, we were happy to take the time for it.”

1. „Es war eine tolle Erfahrung und es war sehr interessant.“ (WP6_KW35_7)

Translation: „It’s been a good experience and very interesting.”

1. „[...]es war super interessant“ (WP6_KW30-3)

Translation: „It was so interesting”

1. „Hat Spaß gemacht, war interessant und man hat sich mal wieder aktiv mit der Thematik auseinander gesetzt.“ (WP6_KW29_5)

Translation: It was fun and interesting, and once again we actively dealt with the topic.

1. „ Vielen Dank für die interessante Studie und die Möglichkeit daran mitzuwirken.“ (WP6_KW28_1)

Translation: „Many thanks for this interesting study and the opportunity to participate.”

1. „Vielen Dank für die herausfordernden Fragebögen und alles Gute für die weitere Forschungsarbeit!“ (WP6_KW29_9)

Translation: „Many thanks for the challenging questionnaires and all the best for further research work!”

1. „Danke für die Belohnung :-) Ich werde mir gerne die Ergebnisse anschauen und danke für die Möglichkeit an der Info-Veranstaltung teil zu nehmen. Wenn Sie weitere Befragungen durchführen möchten, können Sie mich gerne anschreiben.“ (W6M-SA104)
   Translation: "Thank you for the reward :-) I will be happy to look at the results and thank you for the opportunity to participate in the info session. If you would like to do any further surveys, please feel free to write to me."
2. „Wir möchten uns für die Möglichkeit bedanken, dass wir teilnehmen durften. Das Ausfüllen war nie langweilig und die Fragen interessant. Gern nehmen wir auch zukünftig an derartigen Umfragen teil, auch wenn wir vielleicht nicht direkt betroffen sind, wie in diesem Fall mit der Allergie unserer Tochter.“ (W6M-KW32_11)

Translation: „We would like to thank you for the opportunity to participate. Filling out the survey was never boring and the questions were interesting. We will gladly take part in such surveys in the future, even if we are not directly affected, as in this case with our daughter's allergy. “

1. „Ja, sehr gerne können Sie mich nächstes Jahr wieder kontaktieren!  Vielen Dank auch für das Informationsblatt und die Links! Ich habe mich gerade schon durch den Allergieinfomationsdienst durchgeklickt - da gibt es ja wirklich sehr viele Infos, toll! Für Alleleland ist unser Kind zwar noch etwas klein, aber zumindest ich hatte gerade schon Spaß daran. In Bezug auf das Informationsblatt wenden wir eigentlich schon alles an; nur der Austausch mit anderen Kindern war in diesem Jahr natürlich etwas beschränkt, was ich sehr schade und kontraproduktiv finde, denn ich denke schon, dass die Neurodermitis bei unserem Sohn auch durch "Langeweile des Immunsystems" bedingt ist (so zumindest mein Bauchgefühl als Biologin). Hoffen wir mal, dass sich daran bald etwas ändert!“ (W6M-CD60)

Translation: „Yes, you are very welcome to contact me again next year! Thank you very much for the information sheet and the links! I've just clicked through the allergy information service - there's really a lot of info there, great! Our child is still a bit small for Alleleland, but at least I've just had fun with it. About the information sheet, we are actually already using everything; only the exchange with other children was of course somewhat limited this year, which I find very unfortunate and counterproductive, because I do think that the neurodermatitis in our son is also caused by "boredom of the immune system" (at least that's my gut feeling as a biologist). Let's hope that something will change soon!"“

1. „Wir würden uns freuen, bald weitere Informationen bzw. auch Ergebnisse der Studie zu erfahren.“ (W6M-HA306)

Translation: “We would be happy to hear more information or even results of the study soon."

1. „Wir sind sehr gespannt, was Ihre Auswertung ergibt und werden die Ergebnisse auf Ihrer Webseite verfolgen.“ (W6M-DC134)

Translation:” We are very excited to see what your evaluation reveals and will be following the results on your website."

1. „Vielen Dank, wir schauen uns das Infomaterial sehr gerne an und sind gespannt zu welchem Ergebnis die Studie kommt.“ (W6M-SA99)

Translation: "Thank you very much, we are very happy to look at the info material and are eager to see the results of the study."

1. „ Die Umfrage teil 3, steht bei mir noch bei ca der Hälfte und ich finde aktuell keine 30min ruhe um dies zu beenden. Ich find den Zeitaufwand für die einzelnen teile leider etwas zu hoch. Mir hätte es in kleineren, dafür mehreren teilen mehr zu gesprochen. Aber ich verstehe natürlich das nicht auf alles Rücksicht genommen werden kann. Ich versuche es diese Woche zu schaffen. Dabei sind mir die 30€. Nicht wichtig.“ (W6M-SA160)

Translation: S253. "The survey part 3, is still outstanding with me at about half and I currently have no 30 min to finish this. I think the time required for the individual parts is unfortunately a bit too high. It would have spoken to me in smaller, but more parts. But I understand of course, that not everything can be taken into account. I'll try to do it this week. The 30€ are not important to me."

1. „ Ich fand die Umfrage sehr interessant. Ich wusste vieles wirklich noch nicht im Detail.“ (W6M-KW45_06)

Translation: "I found the survey very interesting. I really didn't know a lot of things in detail.”
