# Supplementary material for: Intentional and actional components of engaged participation in public health research studies: qualitative synthesis of a recruitment and retention process into the theory-informed INTACT-RS framework
Source: BMC Med Res Methodol. 2023 Jan 16;23:17. doi: 10.1186/s12874-023-01838-3 (PMC9841138; doi:10.1186/s12874-023-01838-3)
Supplement: Supplementary file 2 — Additional file 2. Full description of further framework components. [file 12874_2023_1838_MOESM2_ESM.docx]

**Supplement 2.** Additional framework components

1) Pre-intention phase

*Attention*

To stimulate awareness of the opportunity to participate in research, we used three broadly approaches to a) individuals: directly and personally approach each individual (e.g., send an email, or call participants from previous research projects; S1-S2; all citations can be seen in supplement 1)), b) organizations: (indirectly) forward a study, followed up with specific direct contact (e.g., asking a midwife to communicate the call for participation to current clients; S3-S7), and c) public: directly but impersonally approach potentially interested individuals (e.g., publish the study call within a radio contribution; S8-S11).

Further, we considered how each invitation’s format and content affect an individual’s or group’s likelihood to participate in research. Regarding content, parents, pediatricians and midwives appeared to be attracted either by specifics (ECAP) or a rather broad (child health) description that resembles their own situation (e.g. parents with an allergic child, parents with no allergies). Thus, to maximize engagement we had explicitly to differentiate our communication of the research topic. In the case of parents for example, those aiming to learn about ECAP were attracted by how the study’s focus is described (S2, S7, S19, S24), while parents interested in taking part in a research project more generally wanted to understand the way the study is conducted (S5). Lastly, options for attracting attention are limited by resources available for recruitment (e.g. time, staff, money). We aimed at balancing between high- and low resource approaches. For the former, we built close personal relations with a limited number of, e.g. childcare facilities, as encouraged, motivated staff would attract more attention compared to, e.g., external researchers (S5, S6, S12, S87, S90). For the latter, we approached multipliers (e.g., childcare umbrella organizations), to spread the call to a larger number of individual facilities, which saved resources. Overall, attention has multiple aspects in terms of how, who and what attracts it. It seems worthwhile to follow and test different approaches in parallel, and, based on initial experiences, continue with that strategy or limit the process to one or two specific strategies, e.g. by considering the relation between resources and outcomes.

Interest

*c) Gratification-oriented*

To attract participants, we used a range of material and intangible incentives (see Table 1). Regarding the latter, few parents stated that financial remuneration constitutes a major motivating factor (study 2a/b), while most did not consider it decisive or even refused to receive an incentive (S38, S62, S68). In the case of financial incentives, one needs to consider when it is precisely effective (e.g. willingness to participate for €30 vs. €50). Multipliers that helped recruit parents considered a €50 remuneration a strong argument, particularly for individuals with lower (socio-)economic status (S69-S71). When recruiting HCPs, monetary gratification was accepted but not considered motivating. Overall, it served to reinforce the reciprocity rule: once study participants receive a favour, they tend to reciprocate this positive gesture. Also, while content- and support-related interests were strong in the case of parents who deal with their child's health, they are less tangible compared to financial remuneration. Hence, the actual value of participation needs to be explicit, particularly when an incentive is below a certain threshold. Besides financial gratification, cognitive activity was found to be an effective motivation: mothers with children aged 0–3 years in particular participated to counteract their self-assessed cognitive underload (S72-S75).

*Appraisal of the appropriateness of the effort*

Besides appraising credibility and utility, participants in each study appeared to make a final judgement during the “pre-intention” phase regarding overall participation effort (S111). Therefore, potential participants need clear information not only in terms of expected time, but also whether different types of effort are relevant. In study 2 and 3, for example, considerable efforts were required to prepare for and be able to participate in written interviews and surveys by those who speak a different language. Overall, a correct and possibly pre-tested estimation of how specific target groups appraise effort remains central in the pre-intention phase, as effort appeared to be a main reason either for deciding not to participate in generally or not showing up to participation appointments (study 2). Further, an effort may rather be considered appropriate when a balance between effort and gratification is created, particularly in those cases with (comparably) high effort (S111-S113) [44]. For instance, simultaneous acquisition of knowledge by working on the questions, or appropriate payment.

2) Planning phase

*Coping/managing self-efficacy regarding expected problems*

As both target groups expressed most concerns about organisation and time, which potentially restricts their willingness to participate (e.g. study 2), they appeared to appraise their own abilities for successful participation. While, therefore, participants’ strategies for handling expected and unexpected problems seem vital when deciding about participation and/or while forming expectations (S102, S139-S142), externally shaping or fostering self-efficacy was challenging (S107, S143-S145). However, in each study, we aimed to at least facilitate self-efficacy estimations/judgments indirectly and to increase confidence by reminding participants that any uncertainty during planning or actual participation can be accounted for by the continuous availability of help and support.

3) Action phase

*Maintenance motivation*

Depending on initial interest formation during pre-intention, maintenance motivation has three focuses: thematic content, supportive and gratification-oriented. While motivation reasons do not differ from the descriptions of thematic-, supportive-, and gratification-related interest, maintenance motivation is more elaborate in the actional phase, as it includes actual participation experiences at this point. Also, positive formative evaluation can initiate a change of interest during participation. Ideally, participants who were initially motivated by gratifications change their interest for reasons of content or support of the study, for instance as they recognize their relevant role and support within the study and receive respective feedback from the study team (S214, S215). We aimed at facilitating a motivational change by preparing interview questions in a way that target groups can relate to, and by maintaining personal contact. However, for participants with a content-wise interest, a change in topic (e.g. knowledge about ECAP vs. knowledge about COVID-19-IP), led to dissatisfaction and reduced motivation to continue (S112, S216, S217).

*Continuing decision*

Following initial evaluation considerations, participants decide about whether to continue participation in the case of follow-up assessments (S224-S226). This decision could be observed for study 3, in which drop-out was highest after completion of part one of the three-staged online survey (total drop-out: 15%). While the reasons for this were diverse, time commitment was mentioned by many as a factor that discouraged continuation (S227-S229). In the case of parents, the decision not to continue participation due to a lack of time was also related back to (missing) resources even for relatively short participation durations (average survey completion: 45 minutes). In study 2a and 4, we aimed to encourage continuation by notifying each parent about the second interview phase and inquiring about their willingness to be re-contacted directly after the initial interview. While initial feedback was positive for almost all participants, a time delay in commencing the second interview round resulted in (only) 57% of participants eventually participating in the follow-up (study 2b).

*Barriers and support factors*

Throughout recruitment and participation, we identified a range of barriers, both in parents (taking care of infants) and HCPs (managing day-to-day healthcare practice), as well as various support factors, particularly, and as indicated above, when potential participants realized/felt an intrinsic motivation and/or interest for participation. These have been grouped into 6 categories entitled: lack of time, technology/equipment, lack of target group specific link, target group-specific uncertainty, skeptical basic attitude, study design (see supplement 2). However, while further specifying and concretizing INTACT-RS by means of the empirical evidence gathered during recruitment and study conduct, it appeared helpful to associate/allocate each barrier and/or support factor to one of the individual framework components to target starting points for action to overcome these barriers. It seemed important to identify in which phase the barriers occur, as they need to be addressed differently. For instance, if technical barriers occur in the pre-intentional phase, motivational self-efficacy may be lacking, In case of technical problems occurring during the intentional phase, support and resources can be used to address and solve them (e.g. study 3: personal contact person serves as technical support). In contrast, fostering coping self-efficacy can be seen as a relevant starting point to overcome this problem during the actional phase. The mapping of the barriers associated with the starting points for action can be accessed in supplement 3.
