# Supplementary material for: Intentional and actional components of engaged participation in public health research studies: qualitative synthesis of a recruitment and retention process into the theory-informed INTACT-RS framework
Source: BMC Med Res Methodol. 2023 Jan 16;23:17. doi: 10.1186/s12874-023-01838-3 (PMC9841138; doi:10.1186/s12874-023-01838-3)
Supplement: Supplementary file 3 — Additional file 3. Identified barriers based on the INTACT-RS framework. [file 12874_2023_1838_MOESM3_ESM.docx]

**Supplement 3.** Identified barriers based on the INTACT-RS framework

| Main barriers | Phase | Definition | Starting point for action |
| --- | --- | --- | --- |
| Lack of time | Pre-intentional | - Low time resources of the target group lead to a general lack of attention to study calls. | Resources (time)  Attention  Interest |
|  | Intentional | - During the planning of the study, the participant becomes aware of his/her limited time resources (e.g. due to upcoming birth, childcare). This lack of resources overrides the existing interest in the study and results in non-participation. | Resources (time, social)  Support by research team) |
|  | Actional | - Limited time resources represent the central cause of early study exit in the context of formative evaluation. | Resources (time)  Maintainance Motivation |
| Technology/ equipment | Pre-intentional | - Fundamental concern that participation would be too technically complex | Motivational self-efficacy |
|  | Intentional | - Lack of technical requirement represents a reason for non-participation (e.g. terminal device, minimum tablet format) . - Digital support services are associated with excessive effort. (e.g., digital accompaniment of questionnaire entry by a personal contact person). | Resources (equipment)  Support by research team |
|  | Actional | - Emerging difficulties with technology during study participation increases premature study exit. - Negative formative evaluation: problem solving requires individual action. | Resources (equipment)  Support by research team  Coping/managing self-efficacy regarding expected problems |
| Lack of target group specific link | Pre-intentional | - Lack of reference to the target group reduces the personal relevance (e.g. allergy prevention for parents without allergies or pediatricians) and the subjectively attributed value/benefit. - As a result, (personal, electronic) study requests are rejected from the beginning. | Appraisal of the study (utility)  Appraisal of the appropriateness of their effort  Interest |
| Target group-specific uncertainty | Actional | - Unpredictable events (e.g. birth, illness of the child, emergency patients) lead to missed appointments (e.g. focus group) or deadlines (questionnaire survey). - Unpredictable events may cause participants to forget appointments or further survey deadlines. | Coping/managing self-efficacy regarding expected problems?  Support by research team |
| Skeptical basic attitude | Pre-intentional | - Concerns regarding the data protection of the study (e.g. recording, saving, evaluating, sharing personal data with third parties (e.g. health insurance), publication of results) can lead to a skeptical attitude and doubt about the seriousness of the study. - Research or call for research is negatively associated with advertising/marketing | Appraisal of the study (Seriosity) |
| Study design | Pre-intentional | - Survey method appears to be a deterrent - Length of questionnaire survey/ interview study is a deterrent - Lack of flexibility in study implementation is a deterrent (e.g., processing of online survey without interruption, long journey for study participation) - Lack of consideration of target group characteristics and needs when conducting the study has an obstructive effect (scheduling e.g. morning appointments for working target group, room planning: no breastfeeding facilities for mothers of newborns) | Appraisal of the appropriateness of their effort |
|  | Intentional |  |  |
|  | Actional |  |  |
